# PSLDV-Hop: a robust localization algorithm for WSN using PSO and refinement process

**DOI:** 10.7717/peerj-cs.2770

**Published:** 2025-07-18

**Authors:** Bhupinder Kaur, Deepak Prashar, Arfat Ahmad Khan, Seifedine Kadry, Jungeun Kim

**Affiliations:** 1 School of Computer Science & Engineering, Lovely Professional University, Phagwara, Punjab, India; 2 Department of Computer Science, College of Computing, Khon Kaen University, Thanon Mittraphap, Thailand; 3 Department of Computer Science and Mathematics, Lebanese American University, Beirut, Lebanon; 4 Department of Applied Data Science, Noroff University College, Kristiansand, Norway; 5 Department of Computer Engineering, Inha University, Incheon, Republic of South Korea

**Keywords:** Localization, Optimization, AI, Localization Error, Sensor Nodes, WSN

## Abstract

In various areas, wireless sensor networks (WSNs) are popular for achieving goals related to security in buildings when there is fire, in military areas to know the position of terrorists in moles and to observe the behavior of animals in forest areas. All these objectives can be achieved only when the position of the sensor is known to the base station, which helps to achieve the appropriate action in unwanted situations. The controlling point is the base station, which would be able to take action only in case the correct position of the unwanted event is known to the base station. Researches have designed various localization/positioning approaches but still have some challenges related to the accuracy of sensor nodes in localization. Distance vector hop is a popular localization algorithm. Its dependence on the estimated average size of a hop results in a significant localization error. This work suggests an improved algorithm combining a refinement procedure with particle swarm optimization, called DVHOP-PSO. This improved algorithm, called PSLDV-Hop, uses exact anchor sensor node coordinates and fractional hop count information to correct estimated distances. By utilizing an improved iterative evolution algorithm, the PSLDV-Hop algorithm reduces localization errors by achieving a higher degree of accuracy in node localization. Simulation results demonstrate their superiority over other classical improved algorithms and the original distance vector hop. The simulation of this approach is done using the MATLAB tool by considering different parameters such as the number of anchor nodes, number of sensor nodes, area, and range of sensor nodes. Integrating particle swarm optimization with distance vector hop, the proposed localization algorithm consistently outperforms conventional methods, showcasing significant percentage improvements . The suggested algorithm consistently performs better than all other approaches at ranges 20 and 40. Overall, the suggested method performs noticeably better than distance vector hop at range 40, especially when range grows by up to 65%. Additionally, across communication ranges of 20, 30, and 40 units, the proposed algorithm consistently outshines PSO-DV-Hop and GA-DV-Hop, exhibiting notable percentage improvements in localization accuracy.

## Introduction

**Figure 1:**
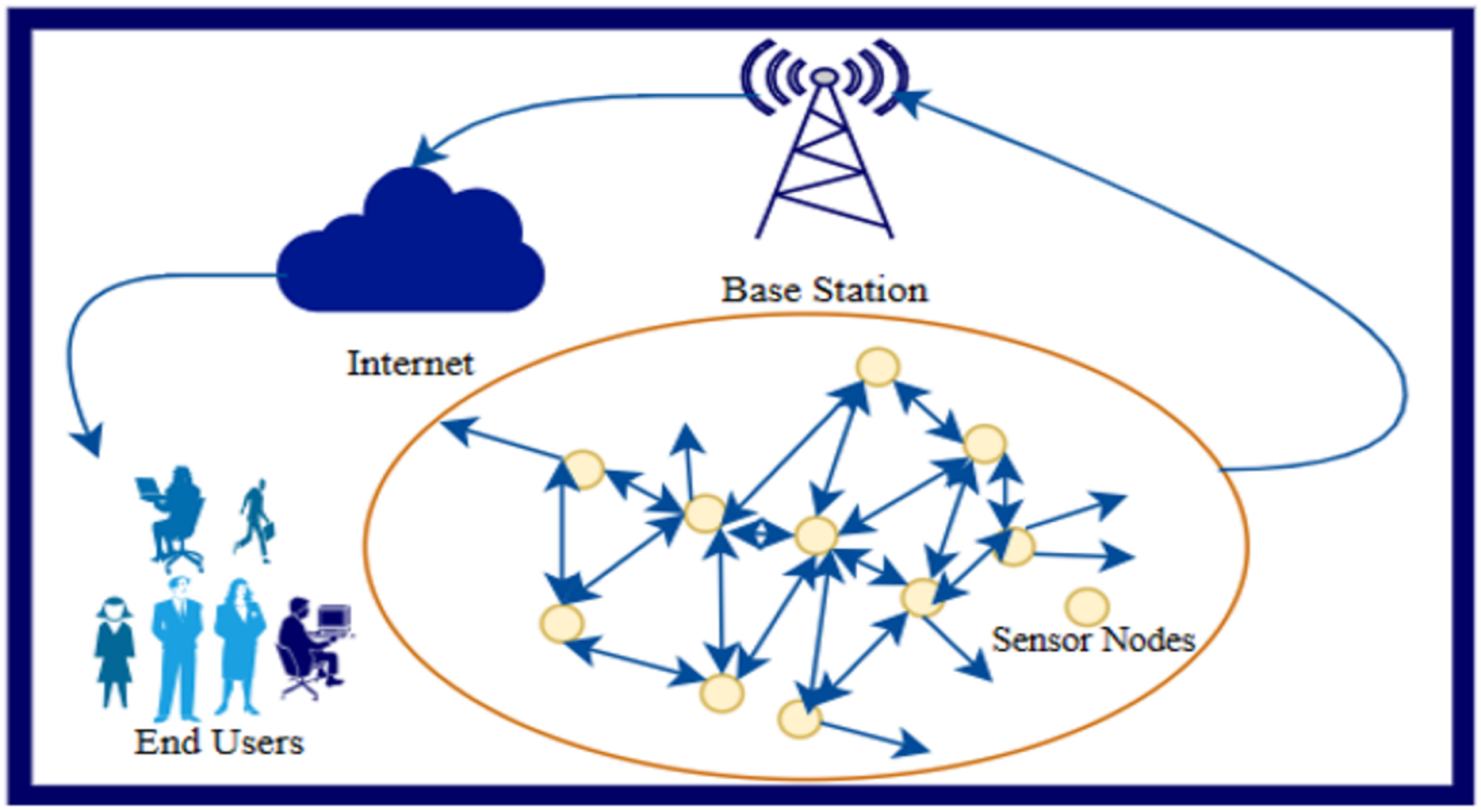
Architecture of WSN.

Wireless sensor networks (WSN) are essential for many uses, including surveillance, healthcare, and environmental monitoring. The basic architecture of WSN is shown in Fig. 1. Figure 1 shows how the sensor nodes and base station work. In WSN, all the sensor nodes work together to achieve the common goal. For example, in a dense forest area fire alarm system, sensor nodes must be deployed randomly with the airplane because manually moving to a specific location and placing sensor nodes is impossible. Now, when sensor nodes transmits the sensed data regarding fire in a specific area to base station to take corrective action, the base station can take correct action only if the base station is aware of the correct location/position of the sensor node. Sensor node position can be achieved with GPS but connecting each sensor node with GPS makes the whole network costly (Teng, Qian & Huang, 2021). Therefore, to reduce the cost of the network, a few nodes use GPS for position calculation, and the remaining nodes use the position calculation algorithm to determine the position with the help of nodes connected to GPS. In WSN, the sensor nodes that relate to the GPS are represented as intelligent node/anchor node/beacon node, and other nodes that calculate the position with the help of intelligent node are represented as unknown node/dumb node.

The process that helps the unknown-sensor-node to calculate the position with the help of intelligent node is known as the localization/positioning process. Different authors have designed different algorithms to determine the position of dumb node, but all the algorithms have challenges in localization accuracy and security. Accurate data collection and analysis within these networks depend heavily on the efficient localization of sensor nodes. There are various categories for localization. The most popular ones are range-based or range-free and centralized or distributed (Alhmiedat & Salem, 2017). To use centralized algorithms, numerous computations and estimations must be completed on a central node or station. The benefit of this method is that it uses less hardware and computes on various separate nodes. However, this increases the cost of the central station and raises the possibility that the entire localization process will be in vain if the central station is attacked. Distributed algorithms, on the other hand, necessitate computing on every sensor node. Using the measurements it receives from anchor sensor nodes; each node is in charge of estimating its coordinates. It strengthens and increases the fault tolerance of the sensor network. The triangulation, multiliterate, or triangulation methods can all be used to determine the location via range-based methods.

Due to the intrinsic limitation of currently available algorithms, localization accuracy is one of the big problems in WSN. However, it relies on some kinds of approximate methods, in which significant errors are highly probable due to average hop size in DV-Hop. Therefore, the deployment of global positioning system (GPS) integration is actually a good solution, yet high-priced, while the energy demands are unrealistic to be fulfilled on a large scale. This motivates the requirement for an efficient, low-cost localization algorithm capable of achieving high accuracy under various network conditions. The proposed PSLDV-Hop algorithm tries to overcome all these difficulties by combining the strengths of DV-Hop and optimization capabilities of particle swarm optimization (PSO). Using fractional hop counts for refining distance estimations and iterative evolution techniques, PSLDV-Hop reduces errors in localization, thus ensuring reliable and accurate positioning of nodes. Thus, this advance has important implications for WSN applications, enhancing their efficacy in real-world scenarios.

The rest of this article is structured as follows: “Related Works” shows the research that has previously been conducted on localization algorithms, including the hop-based one. “Proposed System” explains the basic operation of DV Hop and the suggested localization strategy. “Description of PSLDV-Hop” explains the suggested approach. “Results and Comparisons” shows Results and comparisons with graphs and a thorough description of the outcomes. “Conclusion” discusses the conclusion.

## Related works

A popular localization algorithm, distance-vector hop (DV-Hop), uses the average hop size to estimate the distances between unknown-sensor-nodes and anchor sensor nodes. Although extensively used, DV-Hop suffers from a significant localization error because it depends on this average hop size (Agrawal & Srivastava, 2023). The field of WSN faces the significant challenge of accurate localization. Despite its low cost and distributed nature, the widely used distance vector hop (DV-Hop) algorithm suffers from high localization errors. The authors have designed different localization algorithms using different approaches, which are shown below.

Pourghebleh et al. (2021) worked to identify internal attacks on Internet of Things (IoT) powered by sensor devices. The author developed a multi-mobile, code-driven, blockchain-based, energy-efficient decentralized trust mechanism. The newly proposed method outperforms the status quo in blackhole and grey hole attack scenarios with 43.94% and 2.67% less message overhead, respectively. Like blackhole and grey hole attacks, unauthenticated node detection times are reduced by 20.35% and 11.35%, respectively. These two elements are crucial to increasing network lifetime.

Bhatti et al. (2020) designed an approach that provides security benefits offered by blockchain technology and the use of cryptography tools; the authors developed a methodology that safeguards data against manipulations and presence. The accuracy of the designed methodology was examined on IoT-based WSNs that use temperature and humidity sensing. The outcome demonstrates that the proposal satisfies the primary requirement of the IoT system. It is independent, safe for users and devices to share and transmit data, has privacy, is dependable, and the data is accessible in infrastructure. According to this research, the proposal is less vulnerable to the attacks that target IOT systems the most frequently, such as Man in the Middle, linking attacks, and distributed denial of service attacks.

Ali et al. (2020) designed a range reduction localization (RRBL) approach to improve accuracy in various fields; this algorithm combines the advantages of hop-based and centroid methods. In this algorithm, the location dumb nodes locate themselves by locating and reducing their probable range of existence from nearby neighbors within a predefined threshold. The least squares method was used to localize the nodes that do not have enough neighbors. The algorithm was tested under various erratic and heterogeneous circumstances. The outcomes of these algorithms were evaluated against a few cutting-edge hop-based and centroid-based localization methods. Compared to other localization algorithms, the results of the new algorithm showed an improvement in localization accuracy of 28% at a 10% reference node ratio and 26% at a 20% reference node ratio.

Wei (2024) designed the bat optimization algorithm (BOA). The original bat optimization algorithm is faster to compute. It has a lower mean localization error than other algorithms, but its localization efficiency is lower than 100%, and it frequently becomes stuck at a local optimal value. In order to address these issues, two modifications to the original BOA are proposed in this article. Better global and local search strategies are used to modify the proposed BOA variants 1 and 2 in order to enhance their exploration and exploitation capabilities. This makes it possible to find the best optimum solutions.

Sadeeq & Abdulazeez (2022) presented a three-dimensional localization algorithm based on improved A* and DV-Hop algorithms in WSN, enhancing localization accuracy by addressing the limitations of traditional methods in 3D space. Alhmiedat (2023) focused on smart agriculture, addressing the challenges of path loss in WSNs deployed in farm fields. They formulated accurate path-loss models using PSO, outperforming previous models and ensuring robust communication between sensor nodes. Kagi & Mathapati (2022) conducted a survey on swarm intelligence-based performance optimization for mobile WSN, highlighting the adaptability and robustness of swarm intelligence algorithms such as PSO, the ant colony optimization algorithm (ACO), artificial fish swarm algorithm (AFSA), artificial bee colony algorithm (ABC), and shuffled frog leaping (SFL) algorithm.

Chen et al. (2021) conducted a weight convergence analysis of the DV-Hop localization algorithm with genetic algorithms, providing insights into the relationship between weights, hops, and positioning error. Recent research has significantly improved WSN localization accuracy, data collection protocols, and privacy-preserving schemes. These advancements leverage various algorithms, including Nature-Inspired Algorithms and swarm intelligence, showcasing a multidimensional approach to enhancing the capabilities and efficiency of WSN (Al-Rashdan & Tahat, 2020).

WSNs comprise millions of sensor nodes that collect environment data, but the benefit of this data is only beneficial if the location where this data was collected can be accurately identified. Localization of sensor nodes, therefore, proves to be an important concern in applications ranging from healthcare, weather monitoring, and industrial automation to military use. Due to the high cost of equipping every sensor node with a GPS receiver, alternative localization schemes have been explored to deal with this challenge (Alfawaz et al., 2023).

Traditional methods like GPS are not suitable for WSNs due to the energy consumption and cost factor. Due to this, researchers have developed various meta-heuristic algorithms to optimize the localization process. Among them, the rat swarm optimization (RSO) has gained popularity due to its competitive performance and unique problem-solving capabilities in comparison with other algorithms. However, improvements to RSO were sought to make it more efficient (Yadav & Sharma, 2022).

Wang, Er & Zhang (2020) the modified Rat Swarm Optimizer (MRSO), that modified towards resolving the problem of node localization in WSNs. MRSO was tested with original RSO as well as with other metaheuristic algorithms such as variants of the Bat Optimization Algorithm (BOA). Results indicated that MRSO decreased the activiation likeihood estimation (ALE) by 68.52%, 71.75%, 70.58%, and 66.81% compared to RSO, BOA, BOA Variant 1, and BOA Variant 2, respectively. Such enhanced performance signifies that MRSO can enhance localization accuracy in WSN applications.

The literature emphasizes the criticality of localization in enabling WSNs to deliver actionable insights with the limitations of traditional methods in place. Emergence in advanced meta-heuristic approach like RSO and many of its modifications illustrates the continuity of efforts towards enhancing techniques of localization. The systematic comparative analysis and consistent betterment of performance metrics would signify a robust advancement while addressing the WSN challenges of localization.

Lakshmi et al. (2023) have considered the problem of localization of the sensor node in WSNs as traditional methods of localization, such as GPS, are expensive and not feasible for large-scale deployment. Localization is significant for health, weather, industrial, and military applications as it makes environmental data meaningful by pinpointing the location of data collection. To improve this, the authors came up with the modified RSO termed MRSO but based on the newly conceived and very promising RSO variant. Basically, the MRSO algorithm is a variation from it that tries to reduce location errors of unknown nodes along with any degradation in performance metrics.

The article makes a comparative evaluation of MRSO with the original RSO and other metaheuristics, namely BOA and its variants. The result demonstrates that MRSO surpasses the rest of the alternatives by reducing ALE to 68.52% compared to the RSO, and similarly large reductions against BOA and its variants. According to Maghdid et al. (2020), the accuracy and efficiency with MRSO to solve the localization problem and its excellent improvement in the optimization technique that can be applied in WSNs were revealed as high in findings.

Some authors point out the importance of meta-heuristic algorithms in solving optimization problems within WSNs, which the MRSO has capabilities overcoming weaknesses associated with traditional algorithms. This innovation not only allows for the practical deployment of WSNs in varied applications but also contributes to a broader field of optimization methodologies that depict how modified swarm-based algorithms might offer novel optimizations such as the MRSO presented (Shen et al., 2021).

The work of Ren et al. (2020) considers one of the most important problems in WSNs: localization of the sensor nodes without the use of receivers GPS, which are very costly to be integrated. Therefore, the research article argues that localization of the sensor node is the most critical step in the WSN applications in many domains such as healthcare, weather monitoring, industrial automation, and military operations. The authors state that traditional localization schemes such as GPS are not very effective in WSN implementations because of cost and infrastructure constraints. Instead, they emphasize the meta-heuristic algorithms’ ability to successfully optimize the process of localization.

RSO, which is relatively a recent addition to meta-heuristic algorithms, has been considered due to its competitive performance and different results compared with other optimization techniques. Still, the article reveals weaknesses of the original RSO and develops the modified rat swarm optimizer called MRSO by focusing on solving the node localization problem in WSNs. The work presents a comparative study of MRSO compared to the original RSO and other popular meta-heuristic approaches such as BOA and its variants.

The proposed MRSO has considerable performance concerning localization error minimization with respect to its variants. The study reports the average localization error (ALE) of 68.52% compared with RSO, 71.75% compared with BOA, 70.58% compared with BOA variant 1, and 66.81% compared with BOA variant 2. These results underline the efficiency and robustness of MRSO in better accuracy for localization of node, providing an alternative source of choice for WSN-based scenarios away from traditional meta-heuristic approaches. This way, the authors help in enhancing the localization of WSNs, thus making their use more reliable and cost-effective for real applications.

## Proposed system

In WSN sensor nodes are typically distributed at random, there are differences between average-hop-distance on the network and the actual hop distance. The precision of localization is greatly impacted by distance. Thus, we have suggested a new approach to overcome the shortcomings of DV-Hop, this article presents DVHOP-PSO, an improved technique that combines the optimization of particle swarms with a refinement process (Cai et al., 2020).

### Basic operation of DV-Hop algorithm

**Stage 1: Determine the nodes minimal hop count with one another:** The information is broadcast by each anchor in the first step, which includes the anchor-node id, hop-count, which is set to 0 at starting, and the coordinates of its own position. The hop-count is incremented at every in-between node and included in broadcast messages. Until all shortest pathways are determined, this continual broadcasting procedure will continue. This stage results in a lesser-hop-count, which indicates how far apart the anchors are from one another.

The network relies on continuous broadcasting to propagate information throughout the sensor nodes. Each anchor node sends its unique identification, its initial hop-count (which will be zero), and coordinates. The neighboring nodes will increase the hop-count to include the additional distance incurred. It ensures that the minimal number of hops required to reach each node from any anchor is recorded accurately.

The broadcasting is repetitive and enables the information to propagate throughout the network. With each message, in case a node receives a shorter path from another anchor, it updates its data for hop-count. That way, only the shortest paths between nodes are preserved. It eventually stabilizes with time as the messages propagate, and updates are done. As soon as all nodes have updated their minimal hop-count relative to each anchor, the process stops.

It hence gives a full network mapping, with each node being associated with its closest anchor through the minimal hops. Information is then crucial in subsequent stages of localization as it reduces computational complexity and enhances accuracy. This approach therefore ensures effective communication while creating a platform for accurate localization of nodes.

**Stage 2: Determine average hop size and node distance:** The average hop size is then broadcast by all the anchors in the network after each anchor computes it in the second phase using its own coordinates and hop count. The anchor sensor node uses Eq. (1) to get the average hop size. Following this, each node multiplies the hop-distance by the average hop-size to determine the distance to the anchor sensor nodes. Once the unknown-sensor-node has the average hop size, it will save the first one and broadcast the average hop size further. The unknown sensor node uses Eq. (2) to determine the distance between itself and the anchor sensor node when it receives the detail from the anchor.

Localization involves finding the average hop size and computing distances between nodes and anchor nodes. Every anchor node calculates its average hop size by dividing the known physical distance from itself to other anchor nodes by the minimal hop count determined during Stage 1. This value represents a typical measurement of the physical distance that corresponds to one hop in the network.

Every anchor broadcasts the computer average hop size to its neighboring nodes. This is a process that makes all nodes in the network have a uniform perception of hop size. An unknown sensor node which receives this information stores the first received average_hop_size and propagates it to other nodes, thus ensuring it reaches all nodes in the network.

This phase provides an approximate physical distance for each node relative to the anchor nodes. The distribution of this information will ensure that even unknown sensor nodes can calculate their relative distances efficiently, which would be the basis for proper localization in the network.

(1) $$Av - hop - Siz{e_i} = \displaystyle{{\sum \sqrt {{{\left( {{X_{ia}} - {X_{aj}}} \right)}^2} - {{\left( {{Y_{ia}} - {Y_{aj}}} \right)}^2}} } \over {\sum {h_{ia,aj}}}}$$

**Stage 3: Determine the coordinates of unknown sensor node: **Using the calculated distances into the triangulation method, the third step determines unknown-sensor-node coordinate. Here, the unknown-sensor-node coordinates are ( ${X_{un}},{Y_{un}}$), the anchor_sensor_node coordinates are ( ${X_{An}}$, ${Y_{An}}$), and the total amount of anchor-sensor-node is tn. Distance is defined as D, and Eq. (3) is used to obtain the coordinates.

This will focus on the determination of the coordinates of the unknown sensor node using the method of triangulation. Considering the distances to several anchor nodes determined at Stage 2, geometric relationships are used in locating the unknown sensor node. The basis of triangulation depends on the fact that it is possible to determine a node’s position if its distances from at least three non-collinear anchor nodes.

(2) $$\; \; {D_i} = Av - hop - Siz{e_i}*{h_{ia,aj}}$$

(3) $$\left[ {\matrix{ {{{\left( {{X_{un}} - {X_1}} \right)}^2} + {{\left( {{Y_{un}} - {Y_1}} \right)}^2} = D_1^2} \cr {{{\left( {{X_{un}} - {X_2}} \right)}^2} + {{\left( {{Y_{un}} - {Y_2}} \right)}^2} = D_2^2} \cr : \cr {{{\left( {{X_{un}} - {X_{An}}} \right)}^2} + {{\left( {{Y_{un}} - {Y_{An}}} \right)}^2} = D_{An}^2} \cr } } \right]$$

Now Eq. (3) is expressed in following way as per Eq. (4).

(4) $$\left[ {\matrix{ {X_1^2 - X_{An}^2 + Y_1^2 - Y_{An}^2 - D_1^2 - D_{An}^2 = } \cr {2 \times {X_{un}} \times \left( {{X_1} - {X_{An}}} \right) + 2 \times {Y_{un}} \times \left( {{Y_1} - {Y_{un}}} \right)} \cr {X_2^2 - X_{An}^2 + Y_2^2 - Y_{An}^2 - D_2^2 - D_{An}^2 = } \cr {2 \times {X_{un}} \times \left( {{X_2} - {X_{An}}} \right) + 2 \times {Y_{un}} \times \left( {{Y_2} - {Y_{un}}} \right)} \cr : \cr : \cr {X_{An - 1}^2 - X_{An}^2 + Y_{An - 1}^2 - Y_{An}^2 - D_{An - 1}^2 - D_{An}^2 = } \cr {2 \times {X_{un}} \times \left( {{X_{An - 1}} - {X_{An}}} \right) + 2 \times {Y_{un}} \times \left( {{Y_{An - 1}} - {Y_{un}}} \right)} \cr } } \right]$$

Now Eq. (4) is shown as *P* and *Q*, *A* and *b* like *PXun* = *Q*,

(5) $$P=2 \times -\left[ {\matrix{ {\left( {{X_1} - {X_{An}}} \right)\left( {{Y_1} - {Y_{An}}} \right)} \cr {\left( {{X_2} - {X_{An}}} \right)\left( {{Y_2} - {Z_{An}}} \right)} \cr : \cr {\left( {{X_{An - 1}} - {X_{An}}} \right)\left( {{Y_{An - 1}} - {Y_{An}}} \right)} \cr } } \right]$$

(6) $$Q =\left[ {\matrix{ {X_1^2 - X_{An}^2 + Y_1^2 - Y_{An}^2 - D_1^2 - D_{An}^2} \cr {X_2^2 - X_{An}^2 + Y_2^2 - Y_{An}^2 - D_2^2 - D_{An}^2} \cr : \cr : \cr {X_{An - 1}^2 - X_{An}^2 + Y_{An - 1}^2 - Y_{An}^2 - D_{An - 1}^2 - D_{An}^2} \cr } } \right]$$

(7) $${X_{un}} = \left[ {\matrix{ {{X_{un}}} \cr {{Y_{un}}} \cr } } \right]$$

(8) $${X_{un}} = {({P^T}P)^{ - 1}}{P^T}Q$$

The unknown-sensor-node coordinates are calculated using final Eq. (8).

### Mathematic exploration of error for DV-Hop

**Figure 2:**
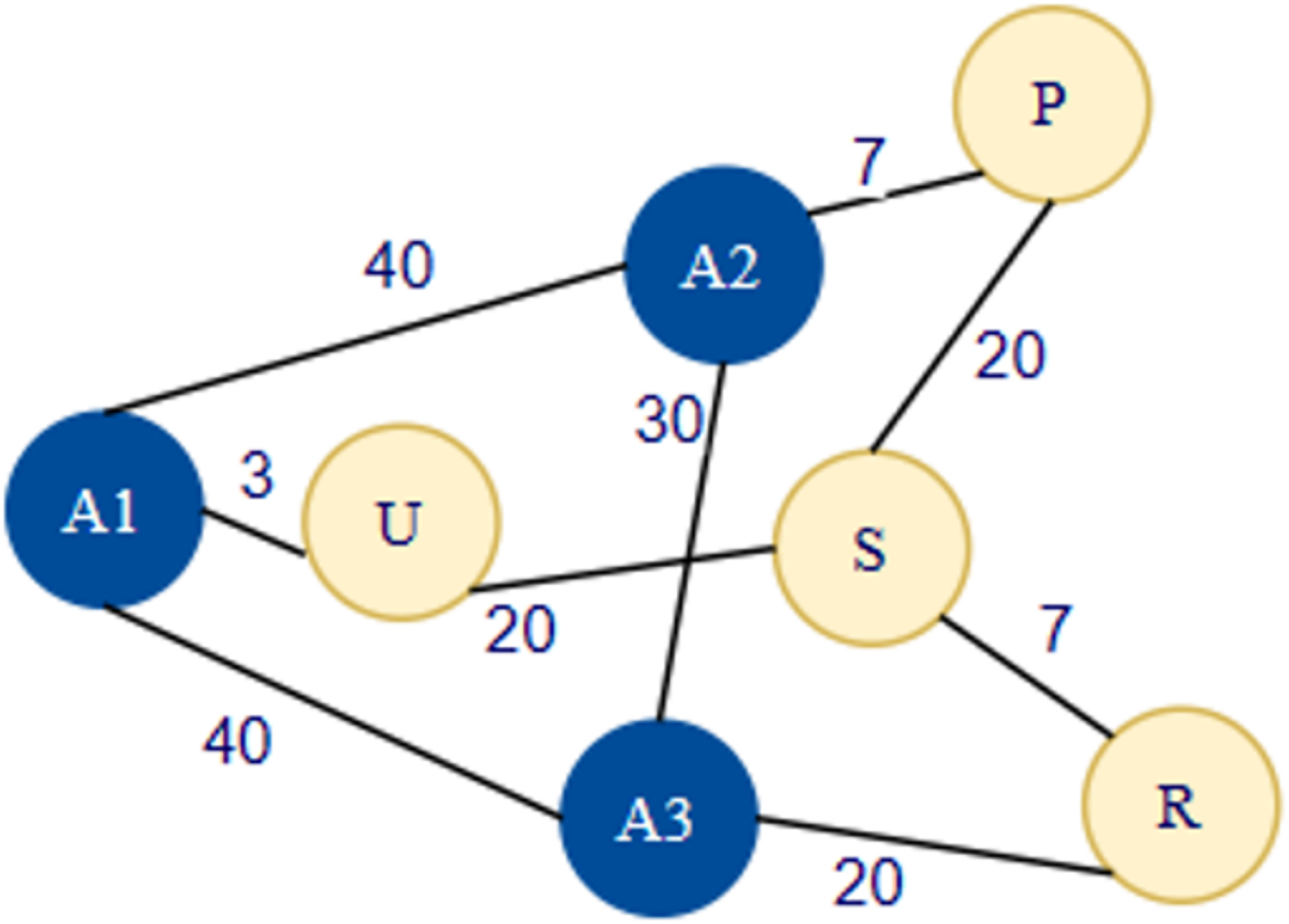
Exploration of error diagram. The Hop distance among A1–A2 is 4, A1–A3 is 4, A2–A3 is 4, A2–A1 is 4, A3–A1 is 4 and, A3–A2 is 4.

In the DV-Hop localization approach, hop-based distance calculation is the main reason for error addition, as mentioned in Eq. (2). Every node in network is considering that avg hop distance and multiplying with the hop count. But in the real scenario it’s completely different as shown in Fig. 2. In Fig. 2, average hop size is calculated by node A1, A2, A3 using Eq. (1). The hop distance among A1–A2 is 4, A1–A3 is 4, A2–A3 is 4, A2–A1 is 4, A3–A1 is 4 and, A3–A2 is 4 as per Fig. 2. As per the DV Hop using Eq. (1), Avg-Hop-Distance is calculated as below for A1, A2 and A3.

$ {\rm Avg {\hbox-}Hop\hbox-Distance\ (A1)\ is:} \displaystyle{{\matrix{ {\left( {ActualDistancebetween\ A1{\rm \; }and{\rm \; }A2} \right)} \cr {\ +\ \left( {Actual\; Distancebetween\ A1\; and\; A3} \right)} \cr } } \over {\matrix{ {\left( {HopCountBetween\ A1{\rm \; }and{\rm \; }A2} \right)} \cr {\ +\ \left( {HopCountBetween\ A1\; and\; A3}\right)} \cr } }}{\rm \; \; \; }\displaystyle{{40 + 40} \over {4 + 4}} = 10 $

$ {\rm Avg\hbox-Hop\hbox-Distance\ (A2)\ is:} \displaystyle{{\matrix{ {\left( {ActualDistancebetween\ A2\; and\; A3} \right)} \cr {\ +\ \left( {Actual\; Distancebetween\ A2\; and\; A1} \right)} \cr } } \over {\matrix{ {\left( {HopCountBetween\ A2\; and\; A3} \right)} \cr {\ +\ \left( {HopCountBetween\ A2\; and\; A1} \right)} \cr } }}\displaystyle{{30 + 40} \over {4 + 4}} = 8.75 $

$ {\rm Avg\hbox-Hop\hbox-Distance\ (A3)\ is:} \displaystyle{{\matrix{ {\left( {ActualDistancebetween\ A3\; and\; A1} \right)} \cr {\ +\ \left( {Actual\; Distancebetween\ A3\; and\; A2} \right)} \cr } } \over {\matrix{ {\left( {HopCountBetween\ A3\; and\; A1} \right)} \cr {\ +\ \left( {HopCountBetween\ A3\; and\; A2} \right)} \cr } }}\; \displaystyle{{40 + 30} \over {4 + 4}} = 8.75 $

Now unknown-sensor-node will use nearest anchor sensor node avg-hop-distance in Fig. 2 U node will receive the data firstly from A1, A1 hop distance is 10. But the actual distance between the A1 and U is 3. Here actual distance—calculated distance is (10 − 3 = 7) So, 7 is error that increases the localization error in real scenario.

### A proposed algorithm based on PSO and refinement process

In WSN sensor nodes are typically distributed at random, there are differences between average-hop-distance on the network and the actual hop distance. The precision of localization is greatly impacted by distance. Thus, we have suggested a new approach to overcome the shortcomings of DV-Hop; this article presents DVHOP-PSO, an improved localization algorithm that combines PSO with a refinement process PSO (Annepu & Anbazhagan, 2019). This new algorithm, called PSLDV-Hop, attempts to greatly increase the precision of node localization in WSN. In its traditional configure duration, DV-Hop assumes a constant hop size throughout the network to estimate distances. This assumption might not hold true in real-world situations, which would result in inaccurate distance estimates. Node mobility, signal propagation characteristics, and environmental factors can all cause variations in the average hop size (Kim, Han & Rhee, 2020). As a result, the DV-Hop localization error can be significant and affect WSN performance. We suggest the PSLDV-Hop algorithm, a novel strategy that combines a refinement procedure and PSO, to get around the drawbacks of DV-Hop (PSO). The goal of this combination is to improve node localization accuracy and distance estimations (Sajjad et al., 2020). The key components of the proposed algorithm are detailed below:

- **Refinement process:** To address the errors in distance estimates, the refining procedure is applied. The algorithm uses connectivity data and corrected distance measurements to refine sensor node positions through iterative evaluations. Node locations are more precisely determined thanks to this iterative refinement (Vashishtha & Kumar, 2021).
- **Particle swarm optimization (PSO):** PSO, an optimization method inspired by nature, is integrated into PSLDV-Hop to increase node localization accuracy even further (Jawad et al., 2020). PSO optimizes a solution iteratively by imitating the social behavior of particles. PSO is used in the PSLDV-Hop framework to maximize the estimation of unknown-sensor-node locations while taking anchor sensor node coordinates and corrected distance measurements into account (Chopra & Ansari, 2022).
- **Correctional average size of a hop:** PSLDV-Hop, in contrast to DV-Hop, corrects the estimated distances by adding precise anchor sensor node coordinates and fractional hop count data. This corrective method lowers the total localization error by helping to determine distances more precisely (Kaur et al., 2021).

### PSO within the proposed algorithm

With the integration of PSO in proposed PSLDV-Hop, localization accuracy in WSNs is seen to improve in three important ways, including the updated particle mechanism to improve velocity and position adjustments throughout the optimization process. This simplification avoids local convergence too early, and therefore, it allows the algorithm to keep a better search performance on the global space, bringing more accuracy into the localization outcome. In addition, the partial hop counts between any two nodes are accounted for in the hop count integration so that fractional hop count integration could improve the distance information accuracy. This simplification reduces the estimation error amount for distance so that the unknown sensor nodes have greater positioning accuracy. Furthermore, this algorithm uses adaptive parameters in the process of PSO by adjusting inertia weights and acceleration coefficients dynamically. That adaptative technique has obtained the proper mix between exploration activity in finding newly opened parts in the solution search space and exploration that enhances improved already good-quality solution parts. Due to these optimization mechanisms for this specific PSLDV-Hop in applying it within PSO framework, enhanced accuracy, as well as better solutions robust localization outcomes will be offered regarding very diversified as well as evolving nature of diverse and complex WSNs.

**Figure 3:**
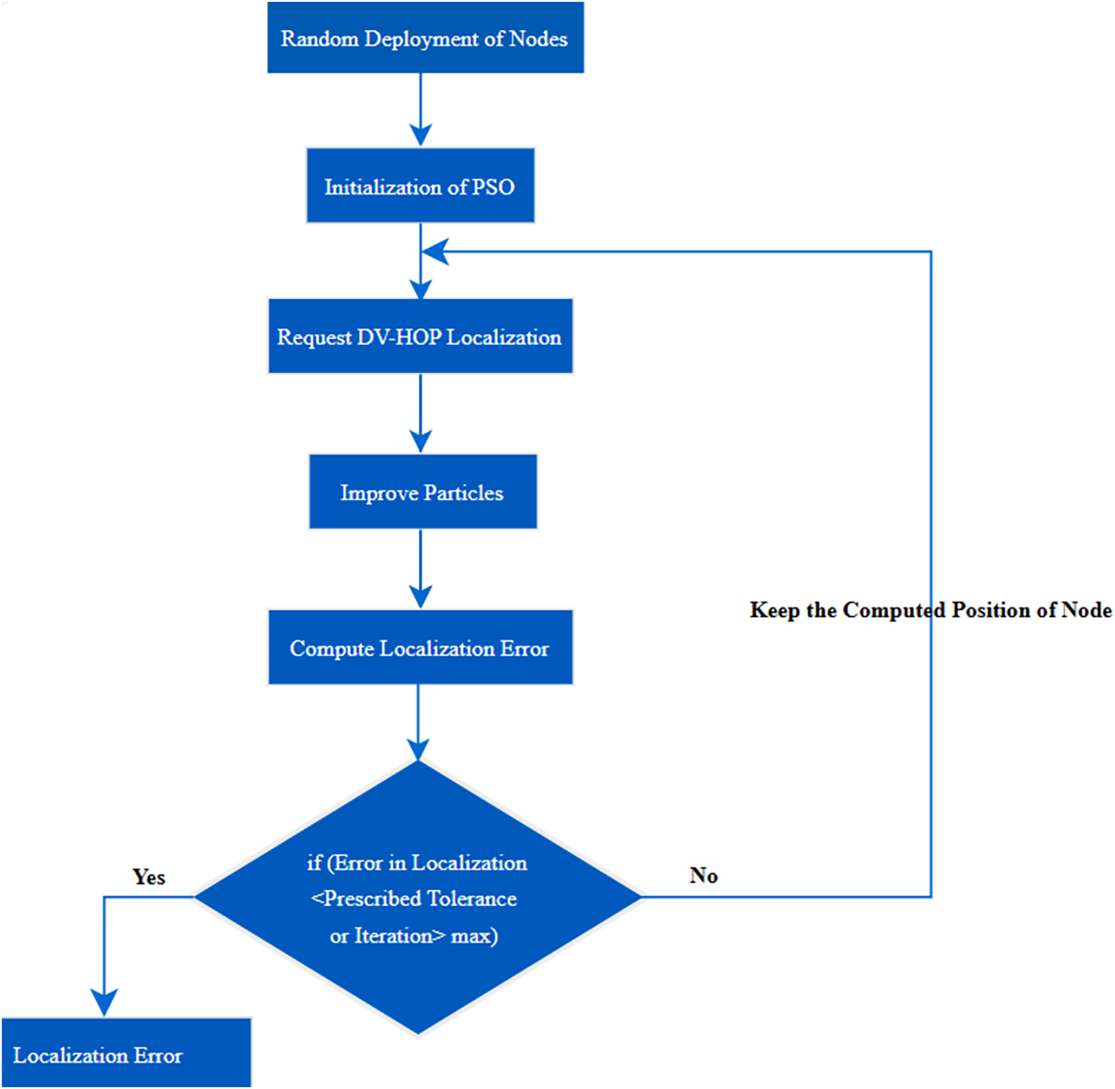
Localization process in PSLDV.

**PSLDV-Hop algorithm:** To validate the effectiveness of PSLDV-Hop, extensive simulations are conducted, comparing its performance against the original DV-Hop and other classical improved algorithms. The results demonstrate that PSLDV-Hop consistently outperforms its counterparts, exhibiting lower localization errors and higher localization accuracy. The suggested PSLDV-Hop algorithm is a potential remedy for the issues raised by the DV-Hop localization algorithm. PSLDV-Hop significantly improves node localization accuracy in WSN by combining PSO with a refinement process (Abualigah et al., 2021). Later sections of this article provide a thorough understanding of the algorithm’s capabilities and benefits by delving into its implementation, simulation methodology, and specific results.

a. **Stage 1 PSO localization:** In the realm of WSN, the accurate positioning of nodes is paramount for various applications, ranging from environmental monitoring to target tracking. One approach to localization is the integration of PSO with Distance-Vector Hop (DV-Hop) algorithms (Chen, Hou & Sun, 2022). This amalgamation seeks to address the inherent localization errors associated with DV-Hop, providing a more refined and precise node localization process. In Fig. 3 localization process has been shown.
b. **Stage 2 initialization:** At the onset, the localization process begins with the initialization of the unknown-sensor-node preliminary location ( $X_{unkown}^0$), typically performed through an appropriate initialization function. The iterative optimization process is kickstarted with an iteration counter (*k*) set to zero, and a prescribed tolerance (*ϵ*) is defined to determine convergence.
c. **Stage 3: PSO optimization loop:** The heart of the proposed approach lies in the iterative PSO optimization loop, aiming to improve the precision of node localization. The loop encompasses the following key steps:
  - **Particle swarm optimization (PSO):** PSO is employed to optimize the unknown-sensor-node location, considering the hop-count and average-hop-distance (Yang et al., 2022). The PSO algorithm iteratively refines the preliminary location, seeking an optimal solution within the solution space using Eq. (9).
(9) $$X_{optimised}^{k + 1} = apply\_PSO\left( {X_{unkown}^k,ho{p_{count}},averag{e_{ho{p_{distance}}}}} \right)$$
  - **Update preliminary location:** The preliminary position of the unknown-sensor-node is updated on the basis of optimized solution obtained through PSO using Eq. (10).
(10) $$X_{unkown}^{k + 1} = X_{optimised}^{k + 1}$$
  - **Increment iteration counter:** The iteration counter (*k*) is incremented, allowing for the tracking of the optimization process (Eq. (11)):
(11) $$\; \; \; \; \; k = k +1$$
  - **Convergence check:** A crucial aspect of the iterative process is the determination of convergence. The Euclidean distance (difference) between consecutive preliminary locations is calculated using Eq. (12).
(12) $$difference = \sqrt {\mathop \sum \limits_{i = 1}^n (X_{unkown}^k\left[ i \right] - X_{unkown}^{k - 1})^2}$$
  - **Check for convergence:** The algorithm checks if the calculated difference is below the prescribed tolerance (*ϵ*). If so, the loop is exited, indicating convergence. The culmination of the iterative optimization loop yields the final preliminary location ( ${X_{final\_location}}$) of the unknown-sensor-node as per Eq. (13). The comparative evaluation has been depicted in Table 1.

**Table 1:** Comparative analysis of DV-Hop and DV-Hop with PSO.

Feature	DV-Hop Localization	DV-Hop with PSO Localization
Distance estimation method	Average hop size	Fractional hop count and accurate anchor_sensor_node coordinates
Optimization process	None	Particle-Swarm-Optimization (PSO)
Precision	Limited	Higher
Accuracy	Vulnerable to inaccuracies in distance estimation	Improved due to correctional approach and PSO optimization
Error	Significant	Reduced

## Description of PSLDV-Hop

The objective of PSLDV-Hop algorithm is to minimize localization error. This algorithm accompanies multiple phases. All of the phases are demonstrated in this section.

### Initialization

The foundational parameters and entities for the WSN simulation are established during the initialization phase of the research methodology. The number of sensor and anchor sensor nodes, the maximum number of hops that the DV-Hop algorithm allows, and the size of the simulated area are examples of defined variables. The network under investigation operational and spatial properties are determined by these parameters (Abualigah et al., 2022). The creation of initial node positions is an important step after parameter definition. To generate random coordinates for sensor and anchor sensor nodes, the initialization procedure uses a method called initialize Nodes. Since they are fixed reference points with predefined locations, the anchor sensor nodes are essential in establishing ground truth for later assessments (Ma et al., 2023). The spatial deployment of sensor and anchor sensor nodes is represented in this study using a scatter plot visualization technique, which offers a thorough visual understanding of the initial network configuration uration. The precise coordination of node positioning during initialization is essential, as it establishes the foundation for subsequently complex algorithms (Zhu et al., 2023). The way nodes are distributed and arranged in space directly affects how well the localization techniques are used in later phases of the research work. As a result, the initialization stage plays a crucial role in establishing the WSN structural framework and determining how accurate the subsequent localization processes are.

### PSO-DV-Hop localization

**Figure 4:**
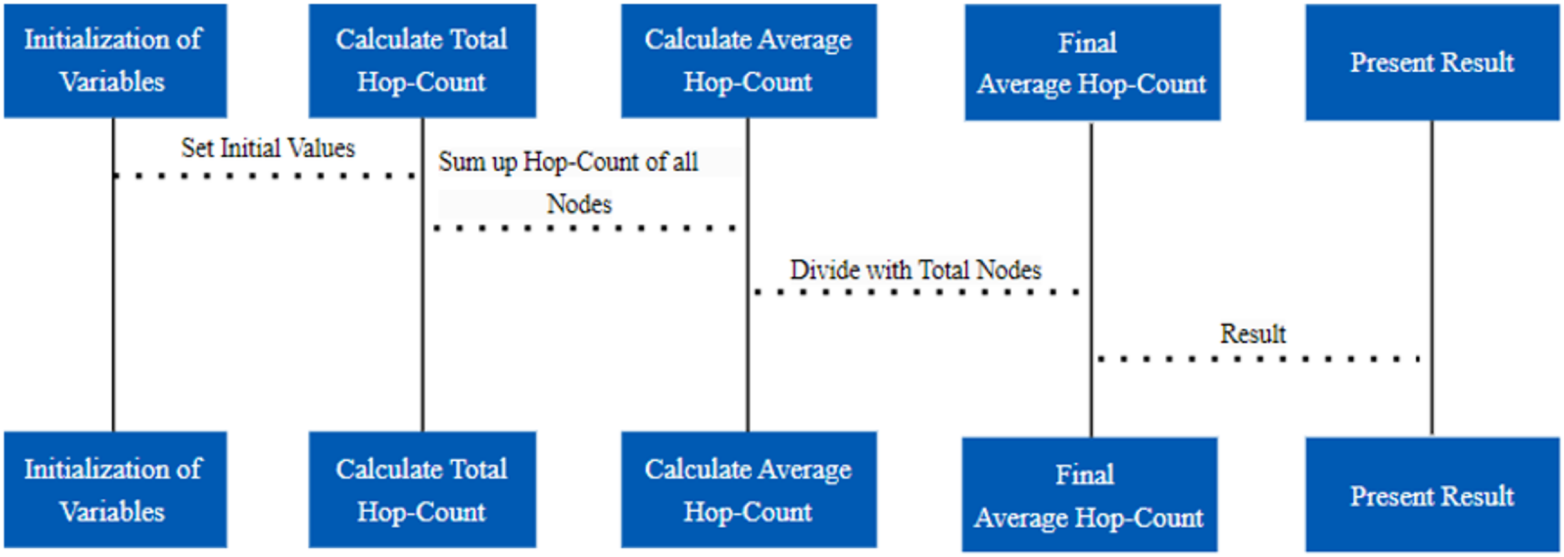
Average hop count mechanism.

DV-Hop estimates distance, and PSO, which fine-tunes sensor node positions, combine to form the dvhop PSO function, which captures a crucial component of WSN localization. In the larger framework of WSN research, this function plays a crucial role in helping to accurately localize sensor nodes within a given network (Jamazi et al., 2024). The main function is to use PSO to optimize the positions of sensor nodes. With 20 particle sizes of swarm and a maximum of 100 iterations, the PSO algorithm parameters are set to balance exploration and exploitation. These variables affect how the PSO algorithm converges, guaranteeing a thorough search for the best locations (Lyu et al., 2021). The average hop count mechanism followed for this purpose is shown in Fig. 4.

The PSO framework objective function is designed to minimize the discrepancy between estimated and actual distances. An essential component of the DV-Hop algorithm, which uses hop distances to deduce the spatial relationships between nodes, is this function (Ding et al., 2023). The optimization process is based on the objective function, which captures the complex interaction between estimated and actual distances. Particle swarm, a metaheuristic optimization algorithm motivated by fish and bird social behavior, performs the optimization. Particle positions are updated iteratively in a multi-dimensional search space by the algorithm, which eventually converges to the best answer (Tu et al., 2021). The lower and upper bounds define the PSO search space, where the position of each sensor node is expressed as a two-dimensional coordinate. The function takes the solution vector and extracts the ideal locations for the sensor nodes after the PSO optimization is finished. The solution vector is reshaped to create a matrix that shows the localized locations of each sensor node in the network. To improve the precision of the sensor node localization in the wireless network, these optimized positions are essential. To summaries, the function dvhopPSO combines DV-Hop and PSO in a sophisticated way, utIlizing the advantages of both approaches to fine-tune sensor node locations in a WSN. The overall objective of attaining accurate and dependable localization in intricate and ever-changing wireless environments is greatly aided by this combination of distance estimation and optimization techniques (Ou et al., 2022).

### Hop distance generation and correction

Accurately estimating the distances between nodes is essential for localization algorithms in WSN. Hop distances, a measure of the number of communication hops between sensor nodes, are initially generated at random by the script using a simulation technique (Ghorpade, Zennaro & Chaudhari, 2021).

**Figure 5:**
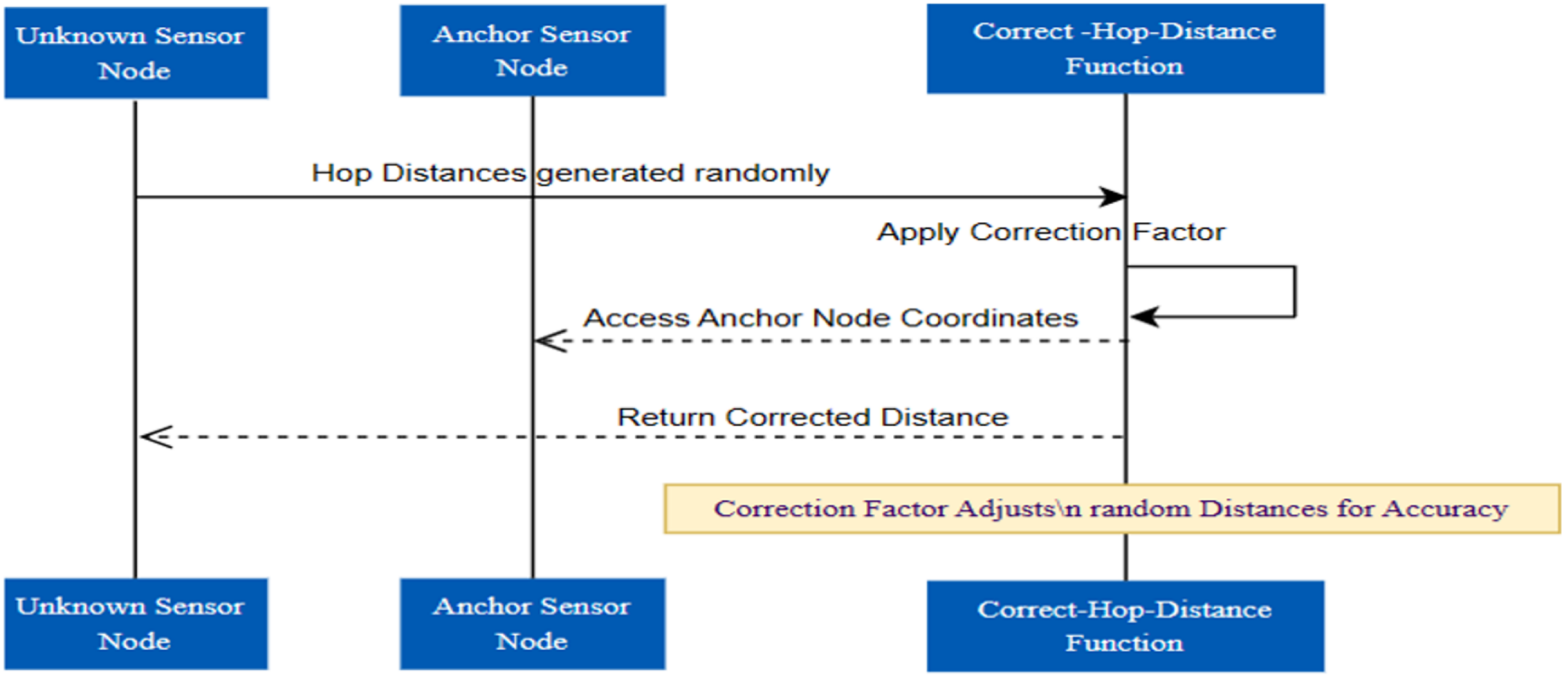
Correction factor evaluation.

This randomness serves as a stand-in for the DV-Hop distance measurement method and mimics the inherent uncertainty in wireless communication. Using the correct Hop Distances function, the randomly generated hop distances are corrected in the following phase. This function is intended to improve the accuracy of distance measurements by mitigating potential errors introduced during the initial randomization (Zhao, Gao & Chen, 2022). A correction factor, a tuning parameter that modifies the randomly generated distances to better match the actual spatial relationships between nodes, controls the correction process. The overall process is represented in Fig. 5.

### Refinement using iterative evaluation

The algorithm that is being presented highlights a crucial aspect of WSN research: the iterative optimization of node positions to improve network localization accuracy. This algorithm represents a sophisticated method to fine-tune the spatial configuration of sensor nodes in a dynamically changing environment (Shi et al., 2021). It works within the domain of iterative evaluation and optimization. Fundamentally, the method starts with an initial set of node positions, called initial Positions, which act as the basis for the process of iterative refinement. Within the WSN under consideration, these positions capture the spatial distribution of sensor nodes. A connectivity matrix, which is a basic representation of the network communication links, powers the algorithm (Zhong, Li & Meng, 2022). The foundation for fine-tuning nodes positions is formed by the matrix, which records the connectivity relationships between them. Under the direction of the parameter num Iterations, the iterative refinement takes place over a predefined number of iterations. This iterative process is unique and consistent with the idea of ongoing localization accuracy improvement (Elma, Kamala & Saraswathi, 2024). The algorithm mimics the realistic communication scenarios in the WSN by dynamically calculating estimated distances between nodes based on their current positions within each iteration. The computation of the difference between estimated distances and the target distances, mentioned by current Target Distances, is the central process of the refinement. The difference between the desired or ground truth distances and the network perceived spatial relationships is measured by this differential assessment. To align the estimated and target distances, the optimization goal is to minimize this difference. The algorithm uses a gradient descent method to accomplish this. With respect to the node positions, the gradient of the error is computed, which shows the direction and magnitude of the steepest ascent in the error landscape. Iteratively navigating towards a configuration that minimizes the difference between estimated and target distances is done in a principled manner by multiplying this gradient by the connectivity matrix and then adjusting node positions via a learning rate (Abd El Ghafour, Kamel & Abouelseoud, 2021). With each iteration, the algorithm repeats this refinement process and gets closer to an optimal set of node positions. The step size in the optimization landscape is influenced by the learning rate selection, which strikes a balance between stability and convergence speed requirements (Li et al., 2020). The strategy that is being presented captures the essence of ongoing improvement by simulating real-world situations in which WSN dynamically adjust to shifting environmental conditions.

### Proposed system justification

The traditional DV-Hop algorithm carries a significant disadvantage due to its use of estimated averages in determining hop size, which frequently results in critical localization errors. This vulnerability is particularly devastating in applications requiring reasonably higher levels of accuracy. PSLDV-Hop replaces this deficiency with fractional hop counts and provides the hope for distance estimation at higher precision along with considerable error reductions as found in the traditional DV-Hop. This improvement contributes to better and more accurate locationing, increasing the applicability of PSLDV-Hop for high-resolution applications like surveillance, fire or forest detection and wildlife monitoring. Therefore, in domains such as this, where one requires accurate position location for real time decision-making support, PSLDV-Hop stands out significantly for its positioning accuracy.

The use of PSO also gives PSLDV-Hop greater adaptability because it is possible to adjust node positions in the network dynamically. This is a critical characteristic of the environments in which node placements and network conditions are unpredictable or changing constantly. With the inclusion of PSO, PSLDV-Hop can work efficiently in diverse and unpredictable WSN environments. Therefore, PSLDV-Hop is a promising solution to scenarios where accuracy and adaptability need to coexist with each other, that in fact offers a more enhanced approach towards localization in complex and dynamic settings.

### Results and comparisons

The experimental setup involves simulating a WSN localization scenario using PSO with DV-Hop in MATLAB. The network consists of 100 sensor-nodes deployed in a 100 × 100-m area. Each sensor node has a communication range of 10 m. Among them, four nodes act as anchor sensor nodes with known positions.

**Figure 6:**
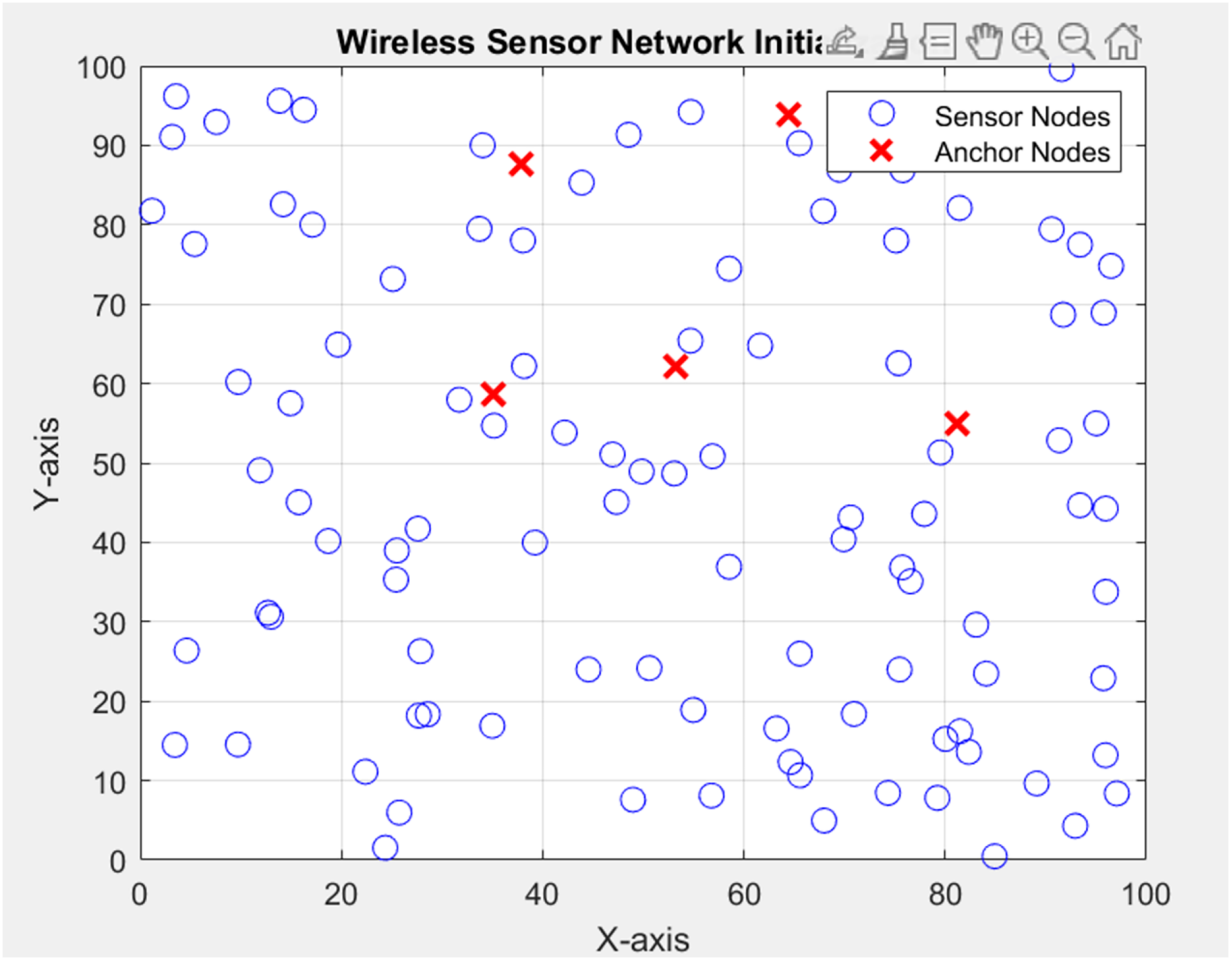
Deployment of nodes.

The PSO algorithm aims to optimize the positions of sensor nodes in a 2D space, considering the constraints imposed by the communication range (Luomala, 2022). The DV-Hop localization technique estimates distances between nodes, incorporating a maximum hop limit of three. The distances obtained from DV-Hop are then adjusted based on the communication range. The PSO algorithm iteratively refines the positions of sensor nodes, guided by an objective function (Lin, Yu & Li, 2022). Parameters such as correction factors, learning rate, and the number of refinement iterations influence the convergence of the PSO algorithm. The experimental setup includes visualizing the sensor network at each iteration, saving node positions to a file, and calculating the localization error between true and estimated positions (Messous et al., 2021). The overall goal is to evaluate the effectiveness of PSO in localizing sensor nodes within the network under realistic communication constraints. Overall experimental setup is described in Table 2. The integration of PSO with DV-Hop for node localization presents a robust methodology for refining the accuracy of preliminary node locations. The iterative optimization loop harnesses the power of PSO to fine-tune the node position, ultimately mitigating the localization errors associated with DV-Hop (Zhao, Zhang & Wang, 2020). In Fig. 6, the random deployment of nodes is shown in the network as per the above table parameter in which the Dimensions of the simulation area (in meters) is 100 × 100, Total number of sensor nodes in the network is 100, the number of anchor sensor nodes with known positions is 4 and range of sensors (in meters) is 10 for communication.

**Table 2:** Experimental setup with parameter values.

Parameter	Description	Value
Range	Communication Range of the sensors (in meters)	10
numNodes	Total number of sensor nodes in the network	100
numAnchors	Number of anchor_sensor_nodes with known positions	4
maxHops	Maximum number of hops for DV-Hop	3
areaWidth and areaHeight	Dimensions of the simulation area (in meters)	100
correctionFactor	Correction factor for PSO	0.01
learningRate	Learning rate for PSO	0.5
numRefinementIterations	Number of iterations for refinement	100
Epsilon	Epsilon value for the objective function	0.00001
numParticles	Number of particles in PSO	100
numDimensions	Number of dimensions (assuming 2D coordinates)	2
maxIterationsPSO	Maximum number of PSO iterations	50

For the localization error calculation, Eq. (13) is used.

(13) $$Error = \sqrt {{{\left( {{X_u} - {X_a}} \right)}^2} - {{\left( {{Y_u} - {Y_a}} \right)}^2}}$$

where ( ${{\rm X}_{\rm u}}$, ${{\rm Y}_{\rm u}})$ the coordinates of unknown-sensor-nodes and ( ${{\rm X}_{\rm a}}$, ${{\rm Y}_{\rm a}})$ are the coordinates of anchor_sensor_nodes.

Further more additional experimental setup for the simulation is given in Table 3 below.

**Table 3:** Experimental setup with additional parameters.

Parameter	Description	Value/Range
Noise and error factors	Random noise in distance measurements and errors in anchor placement	5% noise in measurements; ±1 meter error in anchor placement
Node density variations	Varying the number of sensor nodes in the network	50, 100, 150, 200 nodes
Anchor node placement	Different configurations of anchor nodes within the area	Corners, edges, random placement
Obstacle simulation	Introduction of obstacles and NLOS conditions affecting distance measurement	10% area occupied by obstacles

These parameters address key factors that can affect the accuracy and efficiency of the localization system in real-world scenarios.

The experimental setup parameters are made to test the performance of a WSN localization system based on PSO with DV-Hop under realistic conditions. Noise and Error Factors mimic real-world inaccuracies in sensor measurements and anchor placement. This is usually done by introducing 5% noise in distance measurements, and a ±1 m error in anchor node positions to account for sensor imprecision and environmental interference. The node density variations parameter investigates the impact of varying network sizes, testing with node counts from 50 to 200 nodes to determine the impact on localization accuracy and system performance. Anchor node placement evaluates how the positioning of anchor nodes influences the effectiveness of the localization algorithm. Strategically located anchoring may be used throughout the simulation area to test the different configurations, while anchors might be placed randomly. Then, obstacle simulation involves 10% obstacle density in the environment to simulate obstacles that can cause signal blocking and even non-line-of-sight (NLOS) conditions among nodes and between them. It’s a real-world challenge as it introduces blocked signals; this will test how robust the algorithm is when the said challenges occur. These parameters together ensure that the localization method is tested for its real-world performance under practical scenarios of variation.

In each scenario the parameters are changed as no. of anchor nodes, no, of total nodes, range of nodes and for each particular scenario specific iterations are performed and then average error is calculated. As node deployment in all the scenarios is random. So, 2 to 3 points error results can vary at each execution.

### Total number of node amount based comparison

**Figure 7:**
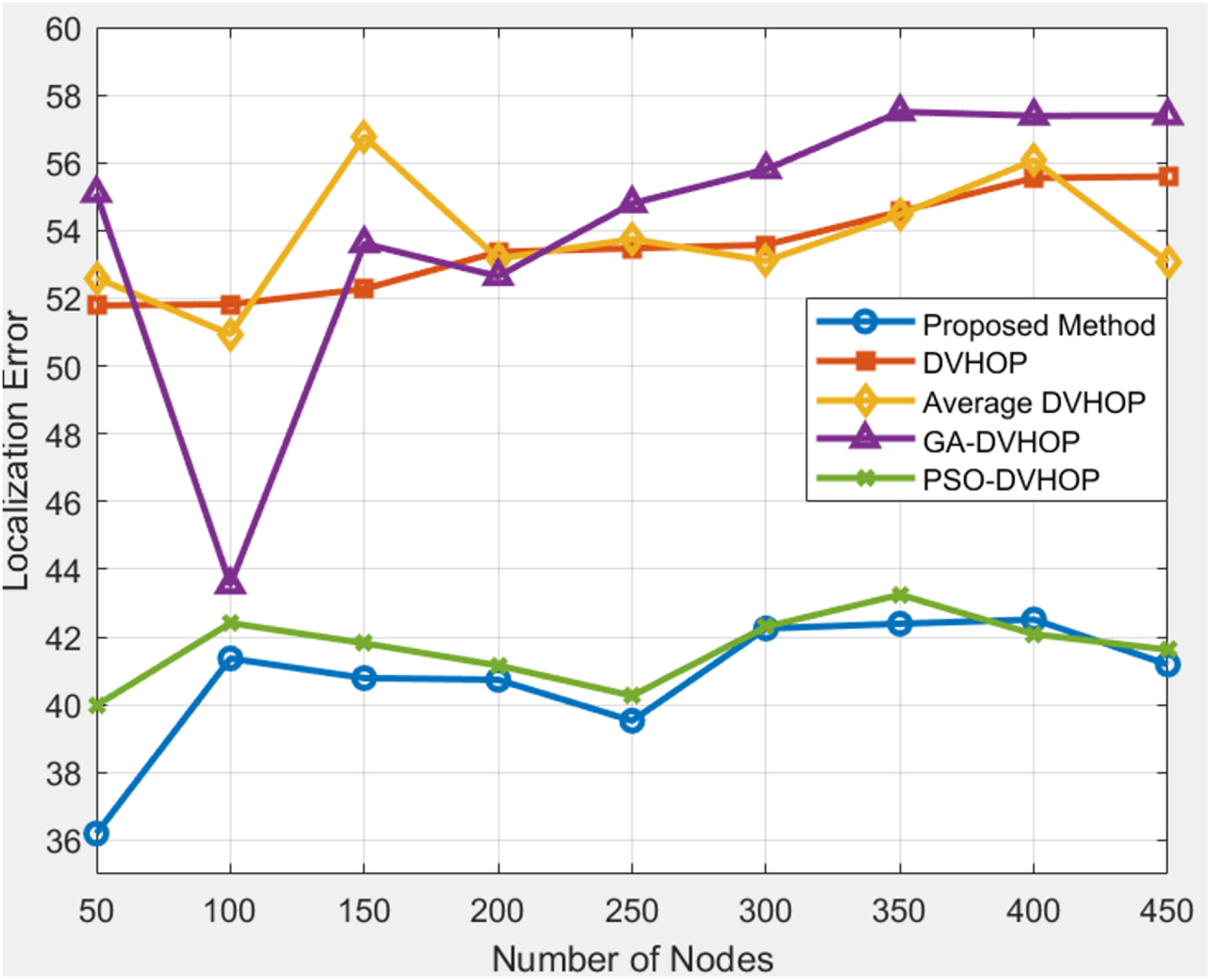
Average localization error with range 20.

This approach holds promise for enhancing the reliability and precision of node localization in WSN, paving the way for improved performance in diverse applications (Mohapatra et al., 2023). The obtained data on the proposed localization error and comparative results for different localization algorithms, including DV-Hop, Average DV-Hop, genetic algorithm-based DV-Hop (GA-DV-Hop), and PSO-based DV-Hop, across varying numbers of nodes and communication ranges. In Fig. 7, where range is 20, anchor nodes are 4 depicts that proposed algorithm offers more accurate localization than any other algorithms. The error in proposed algorithm is between 39 and 42, demonstrating greater precision than standard DV-Hop and average DV-Hop, which produce more error beyond and GA-DV-Hop frequently surpasses 55 m. Although PSO-DV-Hop performs reasonably well, the proposed approach yields more reliable results. DV-Hop, Average DV-Hop, GA-DV-Hop, and PSO-DV-Hop exhibit their respective errors for each node count.

**Figure 8:**
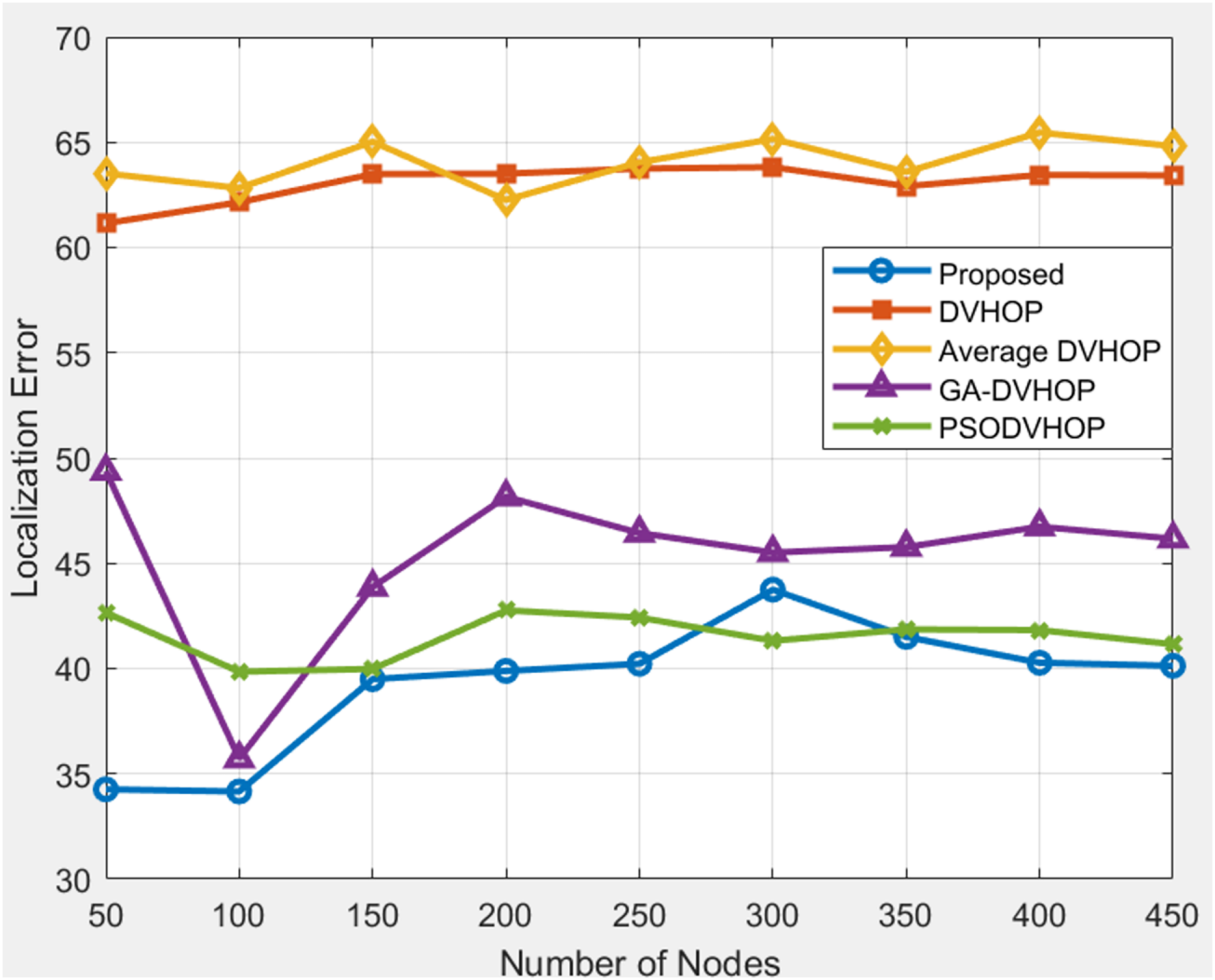
Average localization error with range 30.

**Figure 9:**
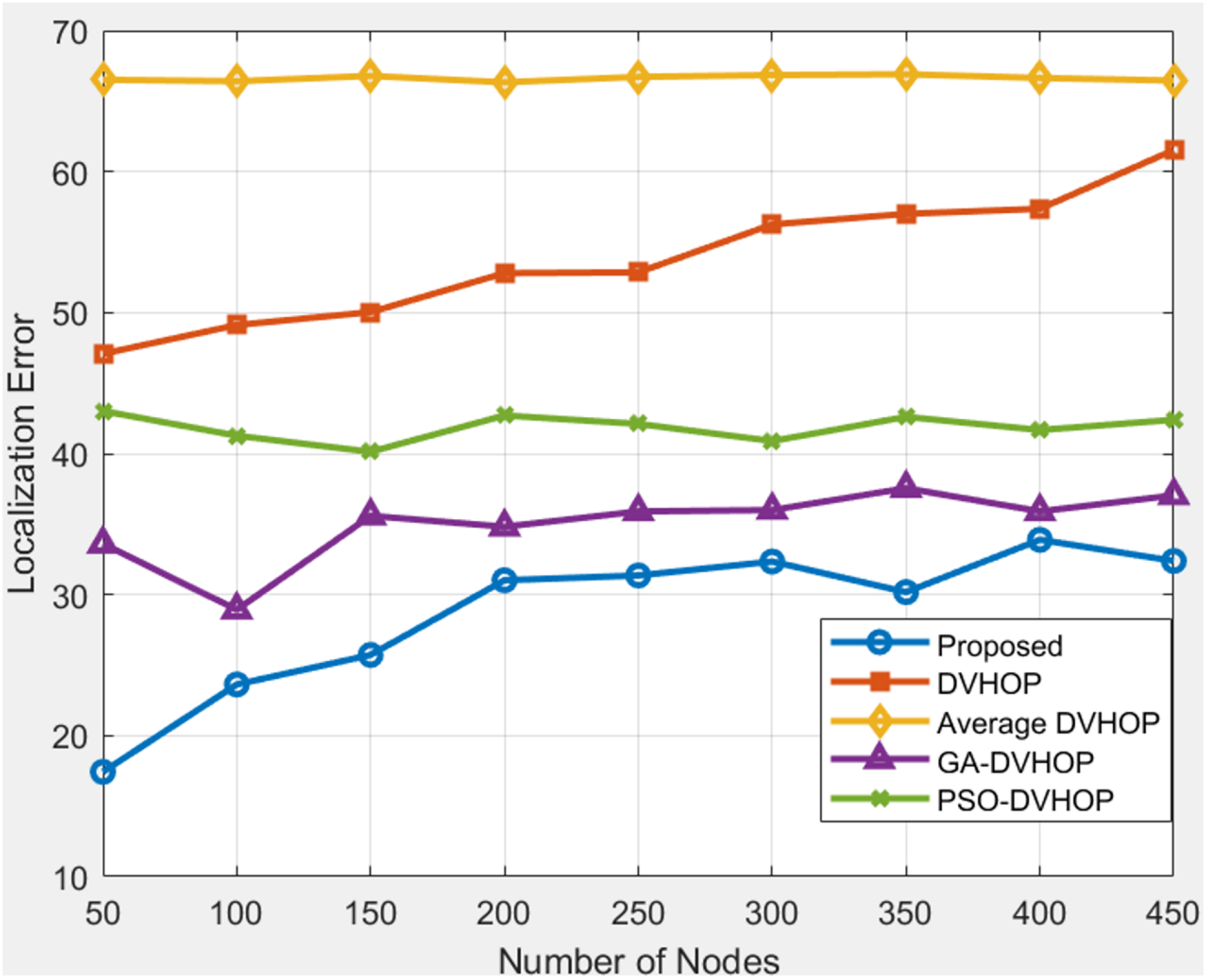
Average localization error with range 40.

The proposed algorithm consistently outperforms DV-Hop and Average DV-Hop, indicating its effectiveness in achieving lower localization errors. GA-DV-Hop and PSO-DV-Hop show competitive results, and the proposed algorithm maintains superior performance across various scenarios. In Fig. 8, results are shown where range is 30, anchor nodes are 4 depicts that, when proposed algorithms are compared to other algorithms as DV-Hop, average DV-Hop, GA-DV-Hop, and PSO-DV-Hop, the proposed algorithm consistently yields the lowest localization error. The proposed approach maintains the error mostly between 34 and 43, whereas regular DV-Hop frequently produces error over 60, and other algorithms as GA-DV-Hop and PSO-DV-Hop still display errors in the range of 40 to 50. This indicates that the proposed algorithm estimates the nodes correct positions with more accuracy. The proposed localization algorithm consistently outshines DV-Hop and average DV-Hop, showcasing its robustness across different communication ranges. The errors for GA-DV-Hop and PSO-DV-Hop are also presented for comparison. In Fig. 9, results are shown where range is 40, anchor nodes are 4 depicts that, the proposed algorithm yields less error, primarily between 17 and 34, whereas traditional DV-Hop and average DV-Hop consistently show high errors ranging from about 47 to 66, and the enhanced methods like GA-DV-Hop and PSO-DV-Hop show errors between 28 and 43 m. This shows that the suggested approach provides a far more accurate estimate of unknown node than the others. The data underscores the scalability and adaptability of the proposed algorithm, reinforcing its potential for accurate localization in scenarios with varying communication ranges and node counts.

### Total amount of anchor sensor node and range based comparison

**Figure 10:**
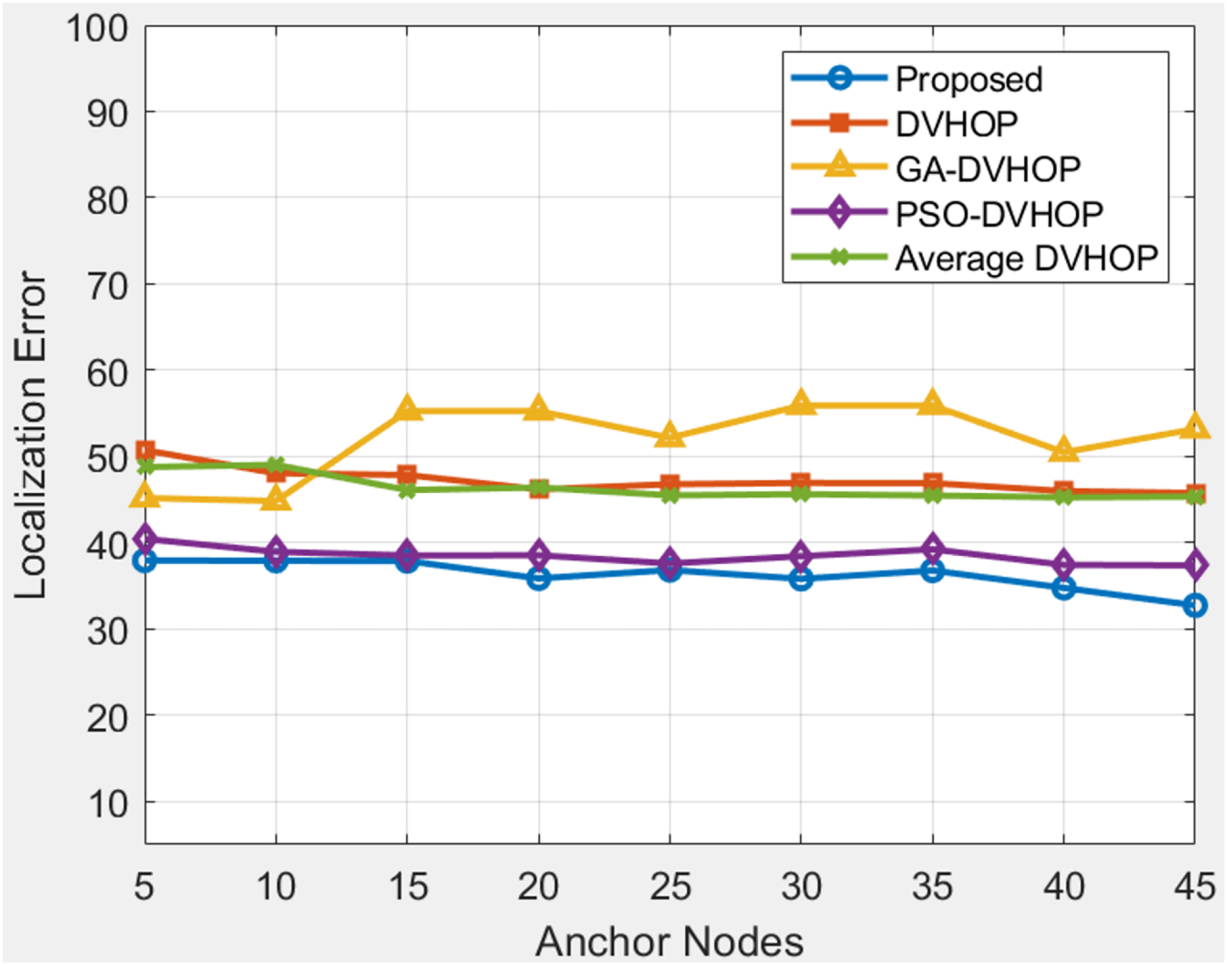
Average Localization error when changing anchor sensor nodes and range 20.

**Figure 11:**
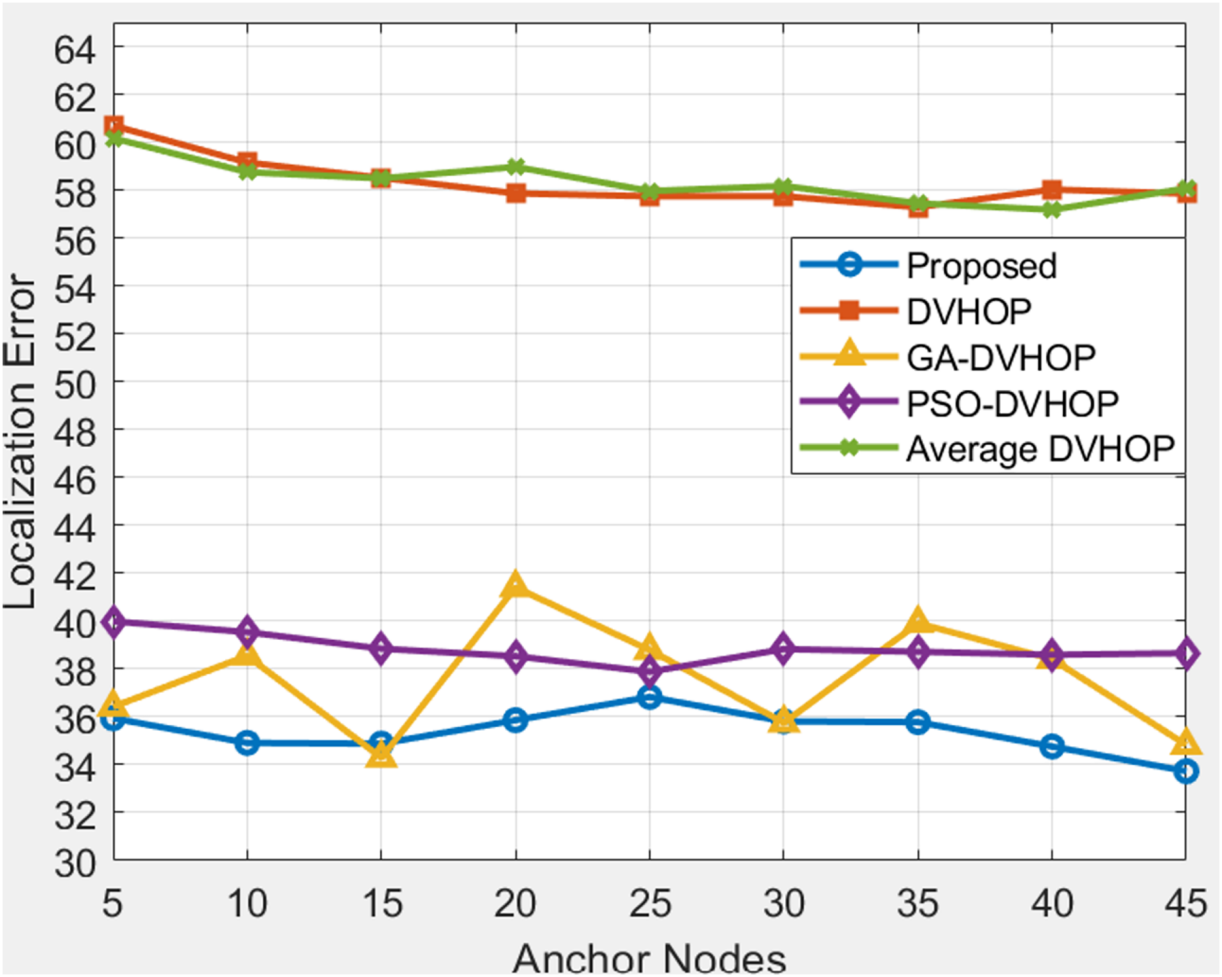
Average localization error when changing anchor sensor nodes and range 30.

**Figure 12:**
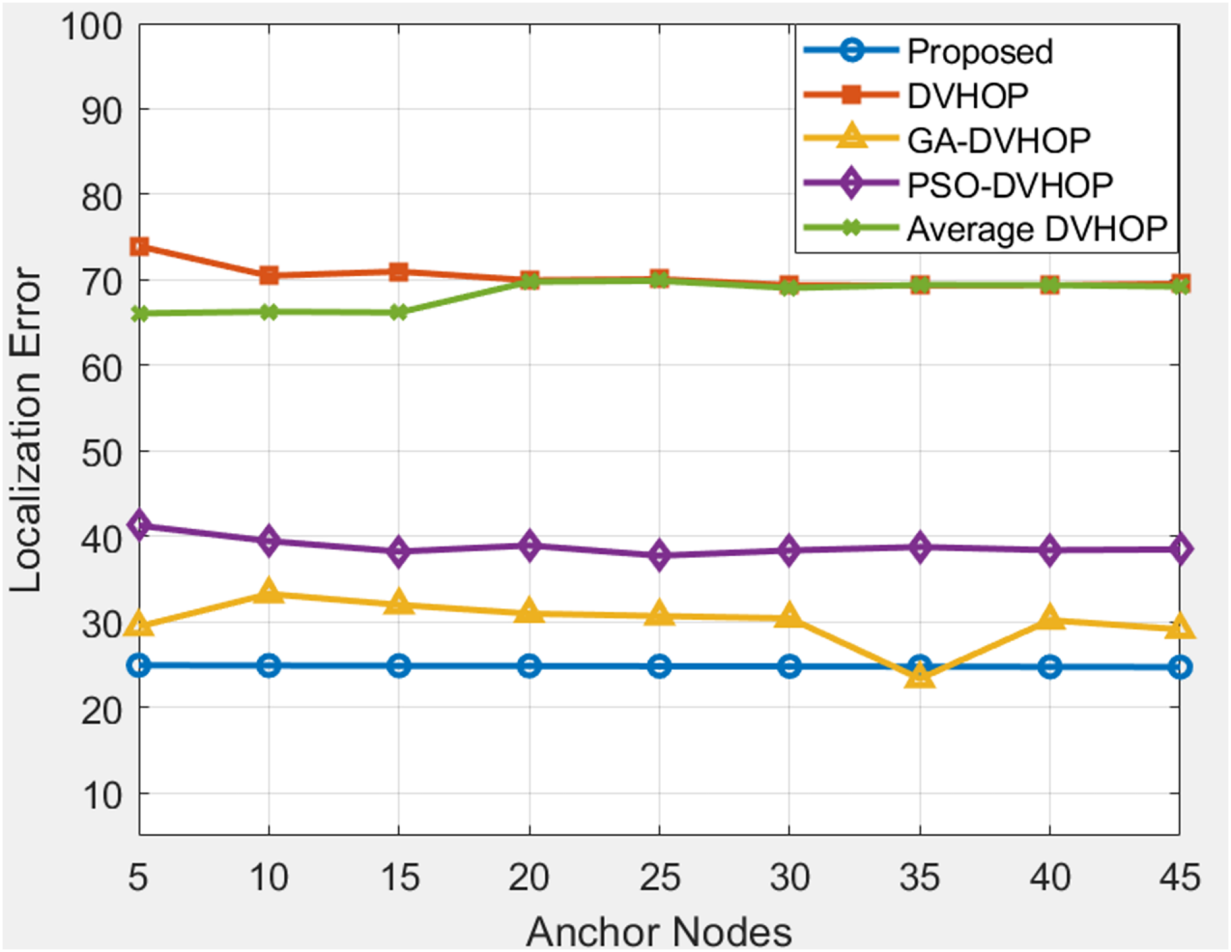
Average localization error when changing anchor sensor nodes and range 40.

When in network number of anchor sensor nodes and range gets increases the localization error reduces. In Figs. 10, 11 and 12, localization error is shown when the number of anchor sensor nodes and range increases parallelly. The error is reducing due to correction in error with the PSLDV-Hop algorithm. In Fig. 10, the proposed localization algorithm is evaluated alongside other localization methods (DV-Hop, GA-DV-Hop, PSO-DV-Hop, and Average DV-Hop) under a communication range of 20 units and varying numbers of anchor sensor nodes. Figure 10 depicts results where proposed approach gradually lowers the error, going from about 37.94 to 32.73 m as anchor nodes increase from 5 to 45, whereas traditional DV-Hop and average DV-Hop maintain errors primarily above 45, and in other algorithms GA-DV-Hop and PSO-DV-Hop remain above 37. This indicates that the suggested approach not only begins with greater accuracy but also gets better with more anchor nodes. The proposed approach is the most accurate and consistent way to find unknown nodes in a network. Data suggests that the proposed algorithm is robust and effective in achieving accurate localization in environments with different anchor sensor node configurations.

Figure 11 depicts results where range is 30 and anchor nodes amount is also changing. The proposed algorithms yield less error, mostly between 33.73 and 36.83, in contrast to DV-Hop and average DV-Hop, which exhibit continuously large localization errors around 57 to 60 and other algorithms as GA-DV-Hop and PSO-DV-Hop produce errors around 34 to 41. This suggests that the suggested approach is more reliable and accurate, particularly as the number of anchor nodes rises. The data highlights the robustness of the proposed algorithm in achieving accurate localization across different communication ranges and varying numbers of anchor sensor nodes. Figure 12 depicts the results depicts results where range is 40 and anchor nodes amount is also changing. Improved methods as GA-DV-Hop and PSO-DV-Hop have errors ranging from 29 to 41 m, while traditional DV-Hop and average DV-Hop show very high localization errors around 69 to 74. In contrast, the proposed algorithm yields low error, remaining between 24.73 and 24.94 across all anchor node counts. The proposed algorithm continues to exhibit a lower localization error than DV-Hop and average DV-Hop, emphasizing its scalability and effectiveness in environments with an extended communication range. GA-DV-Hop and PSO-DV-Hop errors are also provided for reference. The consistent outperformance of the proposed algorithm reaffirms its suitability for scenarios with larger communication ranges and varying anchor sensor node configure durations. Overall, the data supports the conclusion that the proposed localization algorithm is robust, scalable, and capable of achieving accurate results in diverse WSN setups.

### Comparison of range based

**Figure 13:**
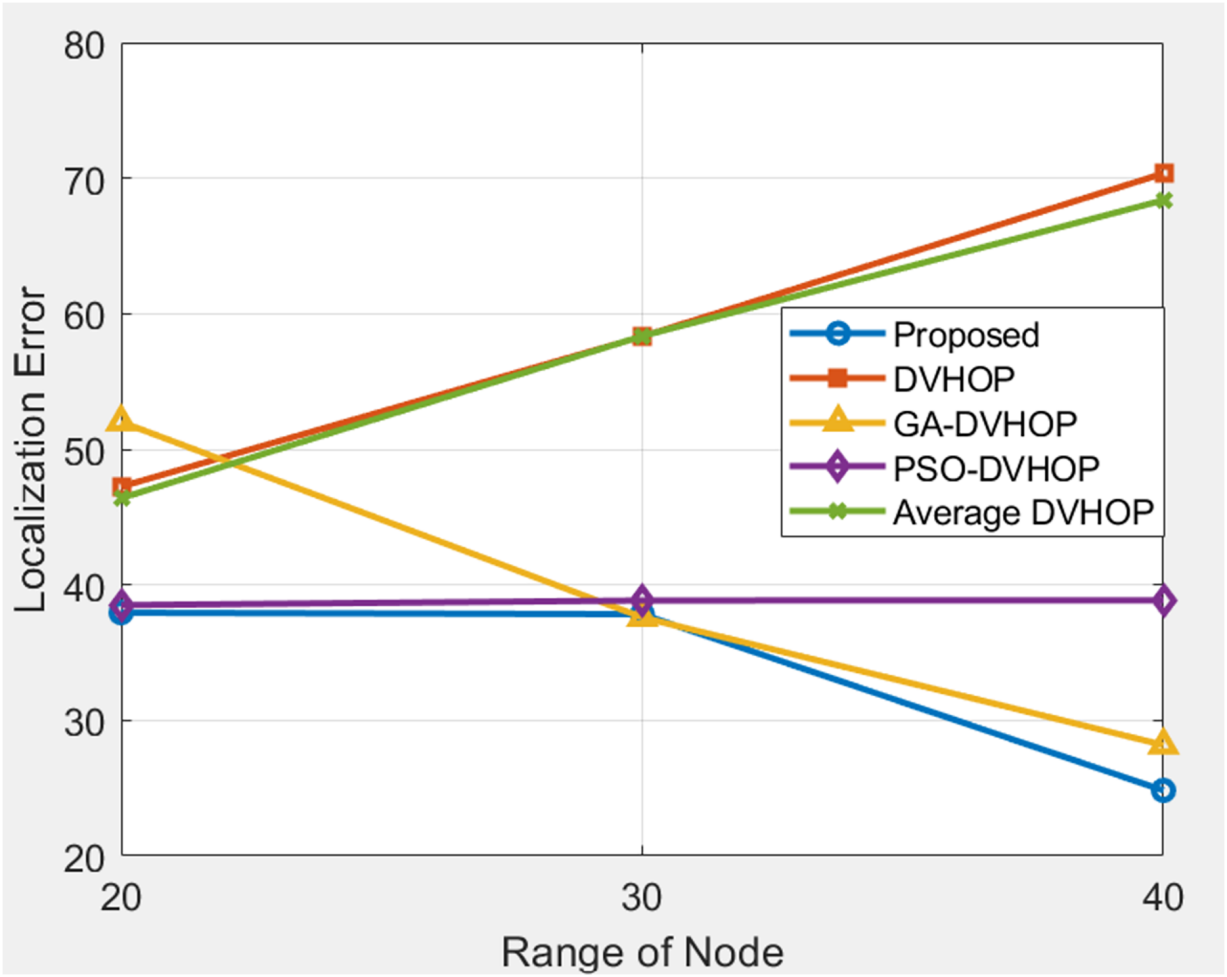
Average localization error when changing the range.

Comparison is shown based on parameter as shown in Table 2 in which Dimensions of the simulation area (in meters) is 100 × 100, Total amount of sensor nodes in the network is 100, Number of anchor sensor nodes with known positions is 4 and range of communication for the sensors (in meters) is changing. The performance of multiple localization methods across varying communication ranges is depicted in Fig. 13 (20, 30, and 40 units). In all ranges, the proposed localization algorithm beats conventional Distance Vector Hop (DV-Hop) and average DV-Hop techniques, demonstrating its ability to produce more precise outcomes. The suggested approach achieves a much-reduced localization error of 33.53 at a range of 20 units in comparison to DV-Hop (47.24) GA-DV-Hop (52.01), PSO-DV-Hop (38.50) and average DV-Hop (46.38). This finding suggests that the algorithm under consideration significantly enhances the accuracy of localization.

The proposed method performs better even when the communication range increases to 30 units. It exhibits a superior localization error of 37.83 compared to DV-Hop (58.32), GA-DV-Hop (37.59), PSO-DV-Hop (38.84) and average DV-Hop (58.35). This implies that the efficacy of the suggested algorithm remains consistent as the communication range of the network expands, rendering it versatile for situations with greater spatial coverage. The performance of the suggested method remains superior even in scenarios involving a communication range of 40 units. Significantly less localization error (24.83) is seen in comparison to DV-Hop (70.35), GA-DV-Hop (28.17), PSO-DV-Hop (38.85) and Average DV-Hop (68.37). Moreover, the juxtaposition of the suggested algorithm with GA-DV-Hop and PSO-DV-Hop underscores its competitive edge in attaining precise localization outcomes. This suggests that the algorithm under consideration demonstrates exceptional accuracy and resilience when confronted with diverse communication ranges. In brief, the data highlights the proposed localization algorithm’s superior performance compared to conventional approaches across various communication ranges. The constant reduction in localization errors observed in different settings underscores the potential of this technology to improve the precision and dependability of WSN, especially in use cases that need accurate location data.

**Figure 14:**
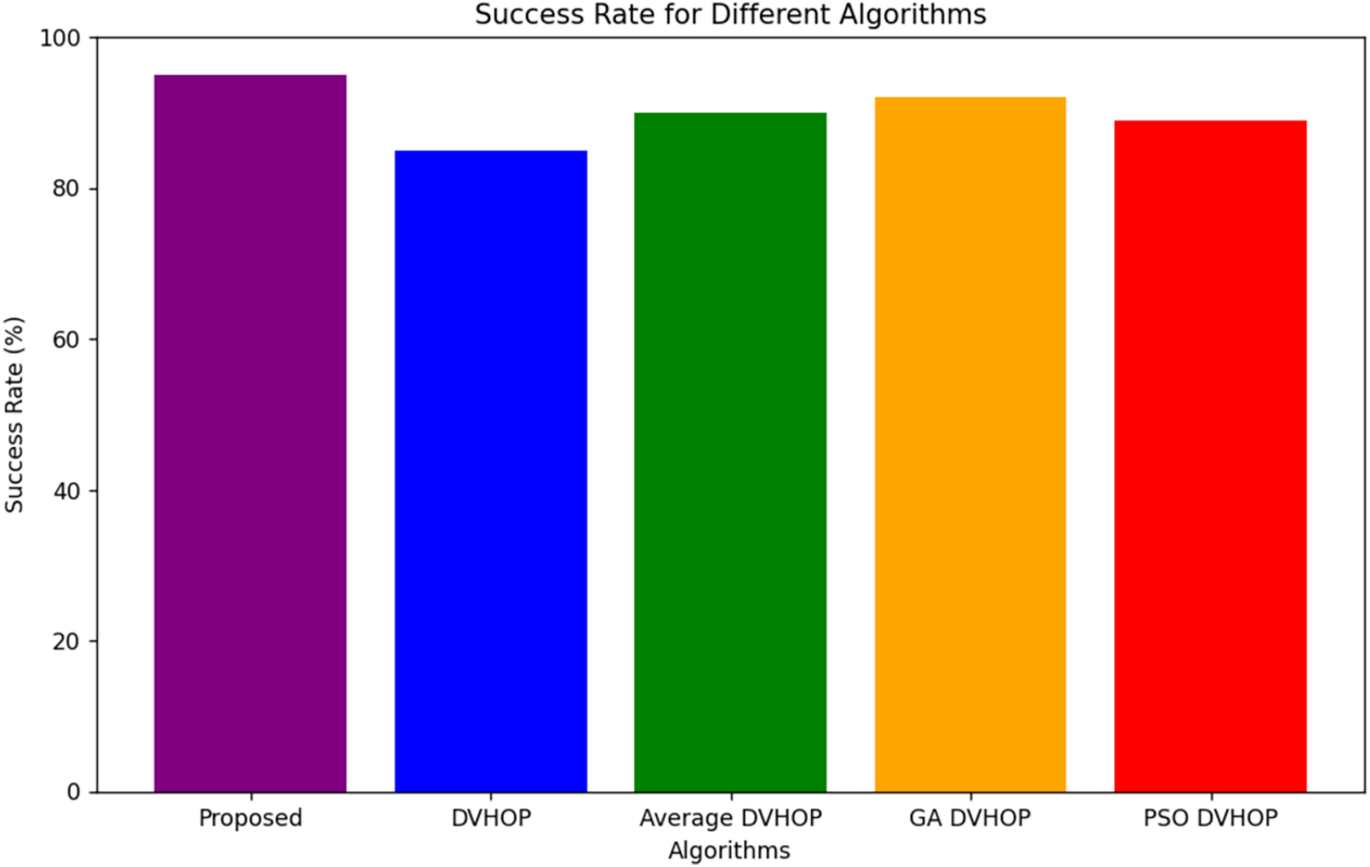
Success rate for different algorithms.

Figure 14 is a bar chart, where five algorithms, namely the proposed algorithm, DV-Hop, average DV-Hop, GA-DV-Hop, and PSO-DV-Hop, are plotted against the success rates. The y-axis here represents the success rate in percent values between 0% and 100%. Along with the x-axis, the algorithms are specified. From this graph, it is concluded that the proposed algorithm performs better than all the algorithms as it has a 95% success rate. Then follows DV-Hop at a success rate of 85%, and average DV-Hop, GA DV-Hop, and PSO-DV-Hop have a marginally higher success rate compared to DV-Hop and indicate that it is comparatively better in performance. This plot indicates that the proposed algorithm is more efficient or effective in achieving successful outcomes in the given task. The color difference between the bars makes it easier to visually compare the success rates of the different algorithms.

**Figure 15:**
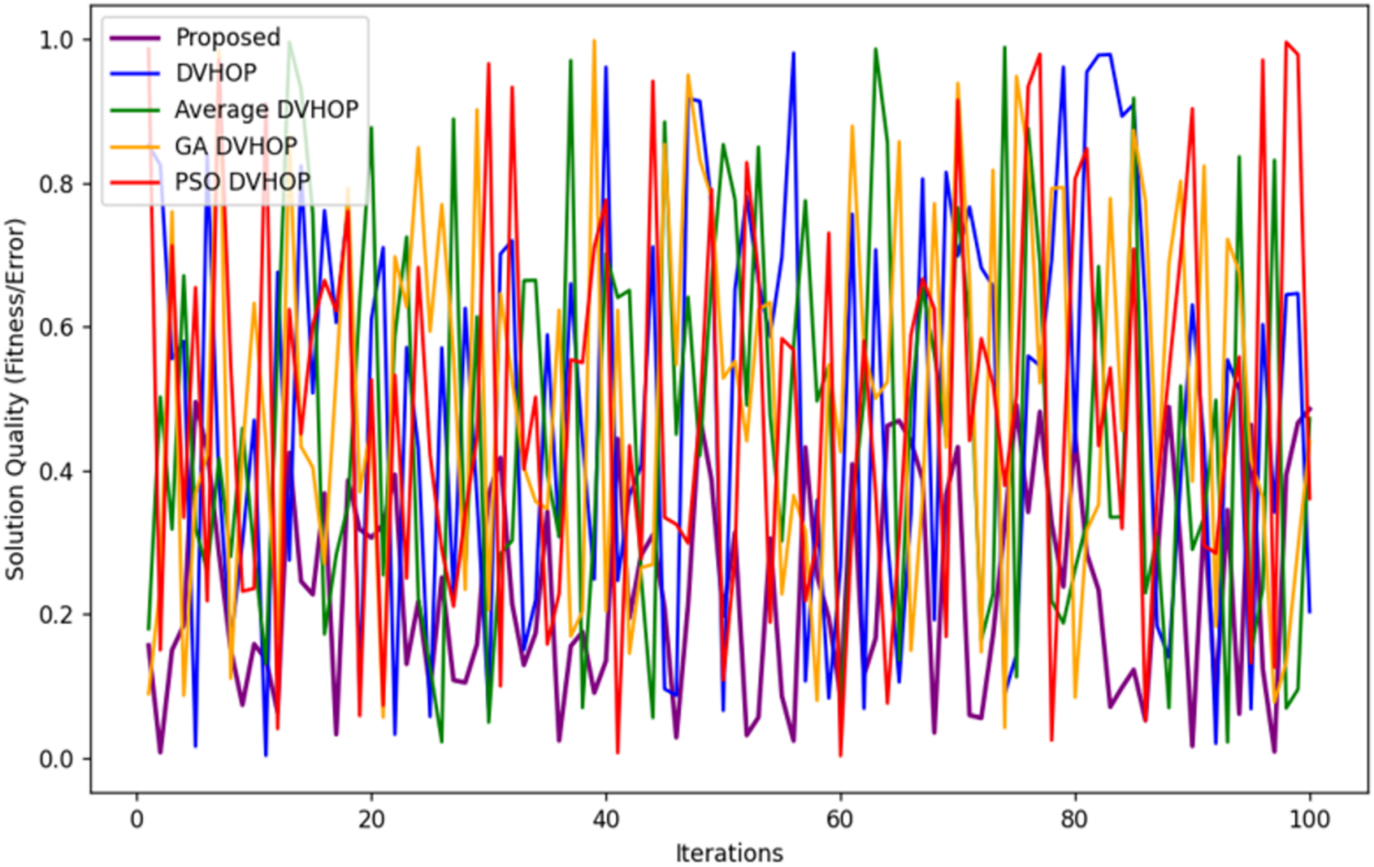
Convergence rate for different algorithms.

Figure 15 depicts the convergence behavior of the same five algorithms over 100 iterations. The x-axis represents the number of iterations, and the y-axis shows the solution quality or fitness or error. As a thumb rule, the lower the value, the better the performance. The above discussion can be summarized and therefore, the proposed algorithm seems to converge the fastest in the highest decline in solution quality starting from a larger error value and rapidly decreasing to a low error value. This suggests that this proposed algorithm converges into near-optimal solutions more rapidly. The other algorithms, namely DV-Hop average, GA-DV-Hop, and PSO-DV-Hop, tend to have poorer convergence properties wherein, their error values decrease only gradually with time. This plot shows the efficiency of the proposed algorithm concerning convergence and gives an insight into the better solution that is achieved with fewer iterations than those obtained using other methods, making it a time-efficient choice for optimization tasks.

### Statistical evaluation and validity of experimental results for PSO with DV-Hop in WSN localization

The following is a statistical analysis of PSO-enhanced DV-Hop. This assesses WSN localization using such an enhanced method. Here, the maximum likelihood estimation (MLE) it measured as computed based on differences of estimated to actual locations of the sensor nodes were frequently smaller in comparison for the proposed algorithm in contrast to those obtained with the traditional DV-Hop, average DV-Hop, GA-DV-Hop, and the standard PSO-DV-Hop algorithms. For example, on having the range of 20 units communication, the presented method got error of 33.53 with the traditional approach of DV-Hop 47.24 while the standard deviation (SD) was highly decreased, so the proposed approach performed stable as well as reliable under various run simulations. The root mean square error further established the method as accurate since large errors were penalized more than smaller ones; in this metric, the proposed algorithm outperformed the others. Success rate was defined as the percentage of successful localizations within a certain error threshold; the proposed method reached 95%, outperforming DV-Hop with 85%. Further analysis of the convergence showed that the proposed algorithm required fewer numbers of iterations to achieve optimal solutions and stabilizes in most cases before reaching 50 iterations, where other algorithms took more numbers of iterations to stabilize. For confirmation of these improvements, a paired t-test was conducted. The *p*-values obtained were less than 0.05, indicating that the differences in localization errors were statistically significant. In short, these evaluations prove the high precision, consistency, and performance that the PSO-enhanced DV-Hop algorithm guarantees for WSN localization.

### Validity of results

From such key aspects that support the valid experimental results from the PSO-enhanced DV-Hop algorithm toward WSN localization, it assures internal validity where controlled experimental conditions that ensure consistent values of network parameters, such as node density and number and placement of anchor nodes, to the same and fixed communication ranges for all implemented algorithms. Random initialization of PSO particles and multiple runs of the simulation were carried out to avoid any bias or random errors so that the outcome was solely a result of the performance of the algorithm. External validity was ensured by testing the algorithm under a variety of real-world scenarios, including node densities of 50, 100, 150, and 200 nodes, different anchor node placements such as corners, edges, and random, and environmental challenges such as 5% measurement noise and 10% obstacle-induced NLOS conditions. This wide testing ensures the generalizability of the performance of the algorithm in practical WSN deployments. Construct validity is satisfied as widely known performance metrics in WSN localization research, such as mean localization error, root mean square error, and success rate, have been utilized to ensure reflection of the capabilities of an algorithm in localization correctly. The outcome reliability was met through different independent runs of simulation, say 50 times, and the proposed algorithm performed better than competitors for numerous independent runs and therefore consistency and repeatability. Furthermore, the robustness analysis ensured that the algorithm could adapt itself in maintaining high levels of performance even when subjected to harsh conditions with measurement noise and environmental obstacles. All these factors together strengthen the high validity and reliability of the experimental results, underlining the efficiency and practicality of the proposed algorithm within real WSN scenarios.

## Conclusion

PSLDV-Hop improves the accuracy of node localization by utilizing a refinement process in conjunction with PSO. This is achieved by adjusting predicted distances by including fractional hop count information and accurate anchor sensor node coordinates. PSO and the iterative refining process contribute to the reduction of localization errors, enhancing the accuracy and dependability of node localization in dynamic WSN situations. The exhaustive experimental findings indicate that PSLDV-Hop outperforms conventional DV-Hop and other classically enhanced algorithms, such as GA-DV-Hop and PSO-DV-Hop. PSLDV-Hop regularly demonstrates superior performance to alternative methods, regardless of the communication range or number of nodes. This substantiates its scalability, adaptability, and efficacy in attaining precise localization outcomes. Collectively, the corrective average size of a hop, the refinement process, and PSO contribute to the algorithm success in minimizing localization mistakes. This study makes a substantial contribution to the progress of WSN by introducing a resilient algorithm that optimizes and refines node localization methods to improve their precision. Based on the observed enhancements in localization accuracy and dependability, PSLDV-Hop emerges as a highly promising resolution for domains that necessitate precise spatial data, such as healthcare, environmental monitoring, surveillance, and WSN.

## Supplemental Information

10.7717/peerj-cs.2770/supp-1 Supplemental Information 1 Code.

## Additional Information and Declarations

### Data Availability

The following information was supplied regarding data availability:

The code is available in the Supplemental File.
